# Timesynth: A Temporal Fidelity Framework for Health Signal Digital Twins

**DOI:** 10.21203/rs.3.rs-10144018/v1

**Published:** 2026-07-01

**Authors:** Md Rakibul Haque, Shireen Elhabian, Warren Woodrich Pettine

**Affiliations:** 1 Scientific Computing and Imaging Institute, University of Utah, Salt Lake City, 84112, UT, USA.; 2 Kahlert School of Computing, University of Utah, Salt Lake City, 84112, UT, USA.; 3 Department of Psychiatry, University of Utah, Salt Lake City, UT, USA.

**Keywords:** Digital Twin, Forecasting Models, Physiological Signals, State Change

## Abstract

Forecasting models for health-signal digital twins must preserve the oscillatory, frequency, phase, and state-transition dynamics of physiological signals, yet the pointwise metrics used to benchmark them cannot detect when these fundamental properties are lost. We show that this blind spot misranks models: across 11 architectures, models with comparable pointwise error diverge by up to **53**° in phase accuracy, equivalent to roughly 123 ms for a 1.2 Hz cardiac rhythm and invisible to standard metrics. To enable development of models that escape such failures, we introduce TimeSynth, a controlled benchmarking framework with two reusable components: a physiologically grounded generator producing signals with analytically known ground-truth dynamics from parametric models fitted to real electroencephalography, electrocardiography and photoplethysmogram signals, along with diagnostics quantifying amplitude, frequency, phase, and state-transition fidelity. Linear and full-sequence attention models systematically lose frequency and phase information despite acceptable amplitude error, whereas architectures with localized temporal structure better preserve dynamical fidelity and adapt to observable state transitions; none, however, reliably preserves stochastic switching. Because the dominant determinant of fidelity is architectural, model choice becomes a principled, use-case-driven decision rather than a search for a single winner. TimeSynth thus supplies the controlled preclinical stress test missing before models are coupled to patient data, with a reusable generator and diagnostics for fidelity-aware development.

## 1 Introduction

Forecasting physiological time series underpins applications from continuous patient monitoring and early warning systems to emerging health-signal digital twins [1–3]: patient-specific computational systems, continuously updated with physiological data, that are expected to predict future biological states [4]. Across these applications, forecasting models must do more than predict future values accurately. Physiological signals encode clinically relevant information in their temporal dynamics, including oscillatory structure, frequency content, phase relationships, and transitions between physiological states that evolve across multiple timescales [5–7]. A model that reproduces future values while distorting these dynamics may appear numerically accurate yet fail to preserve the physiological behavior it is meant to represent.

Yet modern forecasting models are typically evaluated with pointwise metrics such as mean squared error (MSE) and mean absolute error (MAE) [8, 9], which quantify numerical agreement between predicted and observed values but reveal little about whether a forecast preserves oscillatory timing, frequency evolution, or phase coherence [9, 10]. Models with similar forecasting error may therefore represent the underlying process very differently: one may reproduce amplitude while drifting in phase, another may preserve dominant frequencies while smoothing clinically meaningful structure. This is compounded because the dynamical properties of real recordings are generally unknown; without ground-truth amplitude, frequency, and phase trajectories, errors from amplitude distortion, frequency drift, and loss of temporal coherence cannot be disentangled [8, 10]. Together, these limitations create a fundamental gap between forecasting accuracy and physiological fidelity.

This gap is particularly consequential for health-signal digital twins. The National Academies of Sciences, Engineering, and Medicine (NASEM) identify verification, validation, and uncertainty quantification (VVUQ) as essential for trust in digital twins [4], and recent reviews note the lack of frameworks for assessing whether predictions preserve physiologically meaningful behavior rather than merely acceptable aggregate accuracy [11, 12]. Clinical data alone cannot settle this: recordings are noisy, nonstationary, and shaped by many interacting processes [13], and events such as arrhythmia onset, seizure evolution, and sleep-stage transitions cannot be systematically controlled, repeated, or repositioned [14]. Real recordings therefore offer limited opportunity to isolate individual dynamical properties from the complexity of physiological variability.

A complementary approach is to evaluate architectures in controlled environments where the relevant dynamical properties are known, a strategy with precedent across biomedical machine learning. Physiologically grounded synthetic generators have been adopted as enabling infrastructure when richly annotated real data are scarce, exemplified by a recent myoelectric digital twin that simulates large, perfectly annotated electromyography datasets to train neural decoders [15]; there, synthetic ground truth is the contribution, not a limitation. In parallel, rigorous benchmarking has repeatedly shown that headline metrics mislead: state-of-the-art cancer drug-response models barely outperform naive baselines once a reproducible, bias-resistant pipeline is imposed [16], and across synthetic electronic-health-record generators no single method is best on all criteria, so generators must be assessed per use case [17]. Existing time-series benchmarks, however, primarily target predictive accuracy and rarely provide explicit ground-truth dynamics; the field therefore lacks a controlled framework for measuring amplitude, frequency, phase, and state-transition fidelity directly rather than inferring it from aggregate error.

**Fig. 1:**
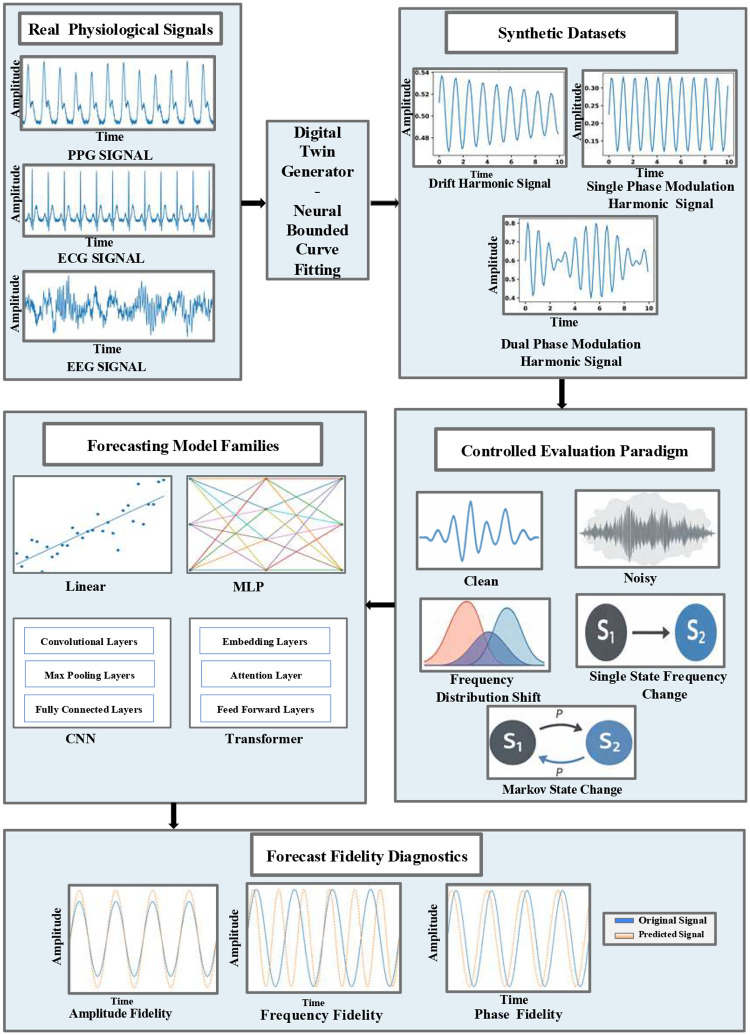
TimeSynth enables controlled evaluation of dynamical fidelity in physiological forecasting. Real physiological recordings are used to fit parametric signal models that generate synthetic time series with analytically known dynamical properties. The resulting signal families provide controlled evaluation environments spanning clean forecasting, noise corruption, frequency distribution shifts, deterministic state transitions, and stochastic switching. Forecasting architectures from multiple model families are evaluated using fidelity diagnostics that separately quantify amplitude, frequency, phase, and state-transition preservation. Together, the signal generator and fidelity diagnostics enable systematic assessment of dynamical fidelity beyond conventional forecasting accuracy metrics.

To address this gap, we introduce TimeSynth, a controlled benchmarking framework for evaluating dynamical fidelity in physiological forecasting (Fig. 1). Synthetic signals are generated from closed-form parametric models fitted to real electrocardiogram (ECG), electroencephalogram (EEG), and photoplethysmography (PPG) recordings, providing analytically known amplitude, instantaneous frequency, and instantaneous phase. The framework applies controlled perturbations, including noise corruption, frequency distribution shifts, deterministic state transitions, and stochastic switching, and evaluates architectures with diagnostics that separately quantify amplitude, frequency, phase, and state-transition fidelity.

Using TimeSynth, we evaluate 11 forecasting architectures across four model families. We show that conventional metrics can substantially misrepresent dynamical fidelity: models with similar prediction error differ by up to 53° in phase accuracy. Architectures with localized temporal processing preserve dynamical structure most consistently (PatchTST [18] and MICN [19] retain phase and frequency fidelity under noise and distribution shift, and PatchTST and ModernTCN [20] adapt fastest to observable state transitions), whereas linear and full-sequence attention models frequently lose phase and frequency information despite competitive pointwise accuracy. No architecture, however, reliably preserves stochastic switching. Together, these findings reveal systematic limitations of current architectures for health-signal digital twins and establish TimeSynth as reusable infrastructure for fidelity-aware evaluation: the controlled, preclinical stress test the VVUQ gap demands before forecasting models are coupled to real patient data.

## 2 Results

We evaluated 11 forecasting architectures spanning four inductive-bias families, comprising linear (Linear, DLinear, FITS), MLP-based (MLinear, NBeats, FreMLP), convolutional and patch-based (MICN, ModernTCN, PatchTST), and full-sequence attention (Transformer, Autoformer) models, as candidate health-signal digital twins. Each was tested under five controlled stress tests that isolate the dynamical demands of physiological forecasting: clean reconstruction, noise corruption, frequency distribution shift, deterministic state transitions, and stochastic Markov switching. The architectural rationale and per-family hypotheses are detailed in Methods. Unless otherwise noted, pairwise comparisons against the linear baseline use paired t-tests with Holm correction; the state-transition analysis uses Wilcoxon signed-rank tests (Methods).

### Physiologically grounded synthetic signals provide controlled ground truth

Real biosignals do not expose ground-truth amplitude, frequency, and phase trajectories, so controlled synthetic signals are required to test whether a forecast preserves these dynamics rather than merely matching future values [5, 7]. We therefore fit closed-form parametric models to recordings from three public datasets and sampled new signals from the resulting empirical parameter distributions, yielding signals whose amplitude, instantaneous frequency, and instantaneous phase are analytically known at every time point (Methods; Supplementary Tables A1–A2).

**Fig. 2:**
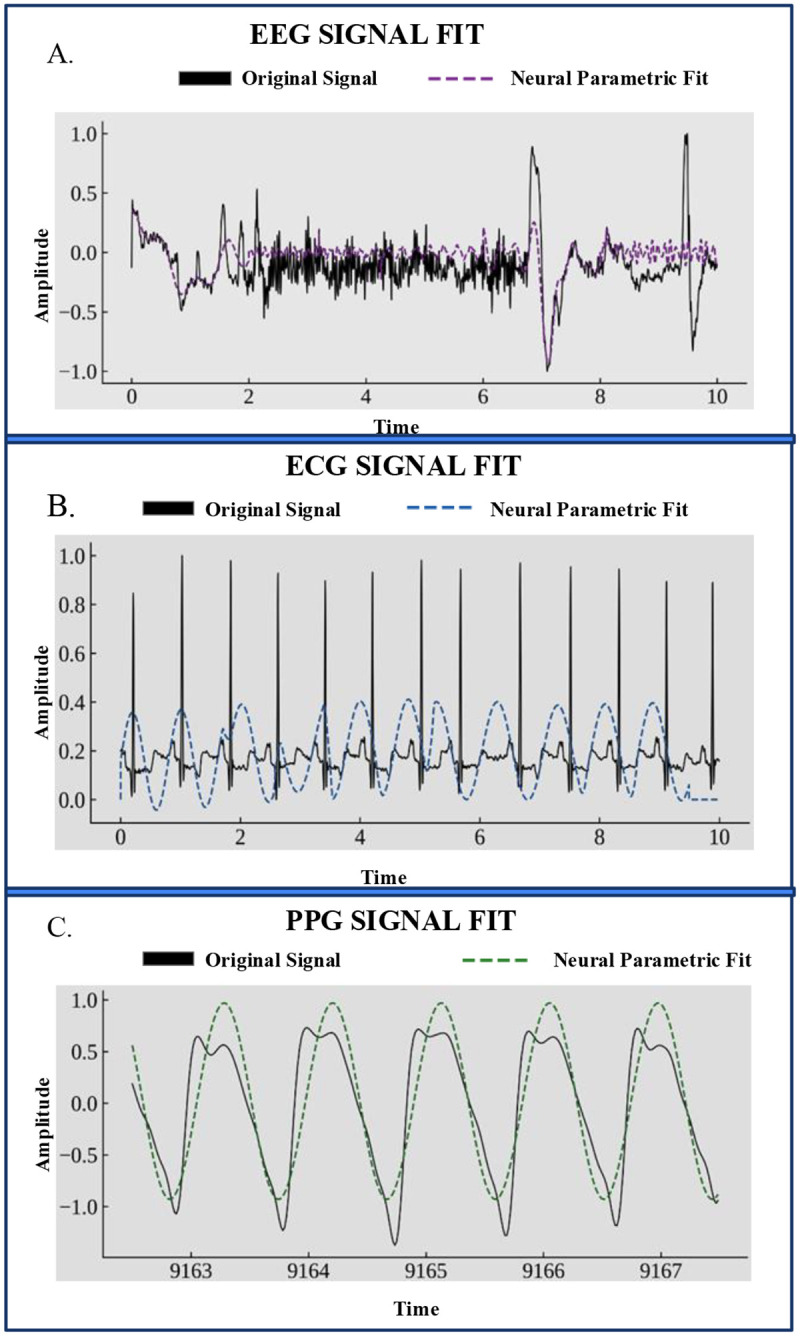
Parametric models fitted to real biosignals ground synthetic evaluation in physiological dynamics. Parametric fits (dashed) overlaid on original recordings (black) for **(A)** EEG (CHB-MIT, channel FP1-F7, 10 s segment), **(B)** ECG (MIT-BIH record 100, 10 s segment, S-Q interval baseline), and **(C)** PPG/BVP (PPG-DaLiA subject S1, best-fit 5 s segment). The fits capture the dominant oscillatory morphology of each signal type while providing closed-form ground truth for amplitude, frequency, and phase.

The fitted families reproduced the dominant morphology of each signal type. The drift-harmonic family, derived from blood-volume-pulse segments in PPG-DaLiA [21], captured the quasi-sinusoidal morphology of peripheral cardiovascular signals, with fitted frequencies concentrated near 1.0–1.1 Hz (consistent with resting heart rate) and near-zero drift coefficients (Fig. 2C). The single and dual phase-modulated families, derived from the MIT-BIH Arrhythmia Database [13, 22], captured the smooth oscillatory baseline of the S-Q interval, excluding sharp R-peak transients that violate sinusoidal decomposition, with carrier frequencies clustered near 1.0 Hz across all 48 records and modulation consistent with heart rate variability (Fig. 2B). The dual phase-modulated family, fit to channel FP1-F7 of the CHB-MIT Scalp EEG Database [23], captured dominant cortical rhythmic activity while accommodating transient deflections (Fig. 2A). Table 1 maps each synthetic paradigm to the physiological phenomenon it approximates, grounding the preclinical evaluation used throughout.

### Pointwise accuracy fails to reflect physiological fidelity

Clinically relevant information in physiological signals is encoded in the timing and rhythm of oscillatory events, not amplitude alone, so a model with low prediction error but mistracked phase or frequency can appear reliable yet fail to preserve the dynamics that motivate a digital twin. Because MSE, MAE, and their normalized variants quantify only pointwise amplitude deviation, we evaluated three complementary fidelity dimensions in parallel: amplitude, phase (timing misalignment), and frequency (dominant-rate mismatch), each defined in Methods.

**Fig. 3:**
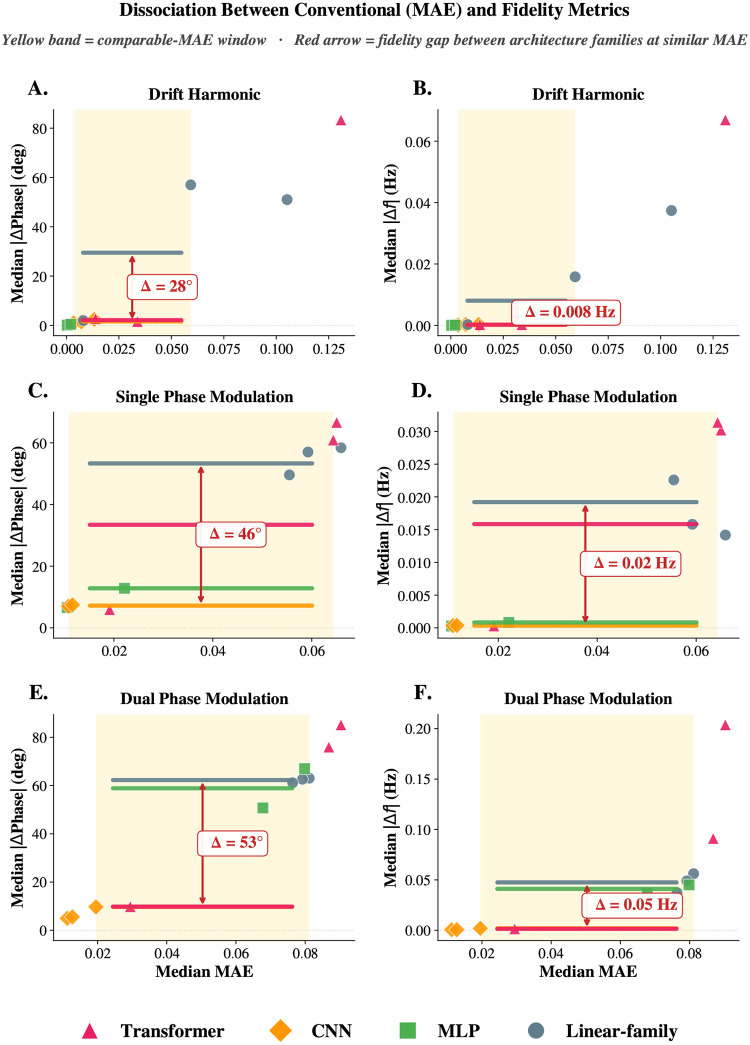
Pointwise accuracy ranks models approximately but cannot resolve phase and frequency differences among models with comparable MAE. Median MAE versus median absolute phase error (left: **a,c,e**) and median absolute frequency error (right: **b,d,f**) across drif-tharmonic (**a,b**), single-phase modulation (**c,d**), and dual-phase modulation (**e,f**) signals. Points are colored by architecture family. The yellow band marks the comparable-MAE window (central 60% of MAE values); horizontal colored lines give each family’s mean fidelity error within that window and red arrows mark the family-level gap. Error metrics are defined in Methods.

Across all 11 models and three signal families, MAE was strongly rank-correlated with phase error (Spearman ρ=0.93,0.90, and 0.97 on drift-harmonic, single-phase, and dual-phase signals; p<0.001 each), confirming that pointwise error captures coarse ordering, yet models with comparable MAE still diverged sharply in fidelity (Fig. 3). Within the comparable-MAE window, linear-family models exhibited phase deviations exceeding 50°, whereas convolutional and patch-based architectures held phase error below 10° (p<0.05, all pairwise). The dissociation widened with signal complexity, from $\Delta =28^{{}^{\circ}}$ on drift-harmonic to 46° on single-phase and 53° on dual-phase signals (about 123 ms at a 1.2 Hz cardiac rhythm), with a parallel frequency gap growing from 0.008 to 0.05 Hz; outside the comparable-MAE window, full-sequence attention models reached frequency deviations up to 0.20 Hz. Pointwise metrics therefore rank models only approximately and cannot resolve the dynamical quantities physiological forecasting requires. The remaining analyses evaluate each fidelity dimension separately across the five stress tests.

### Localized architectures preserve oscillatory structure on clean signals

**Fig. 4:**
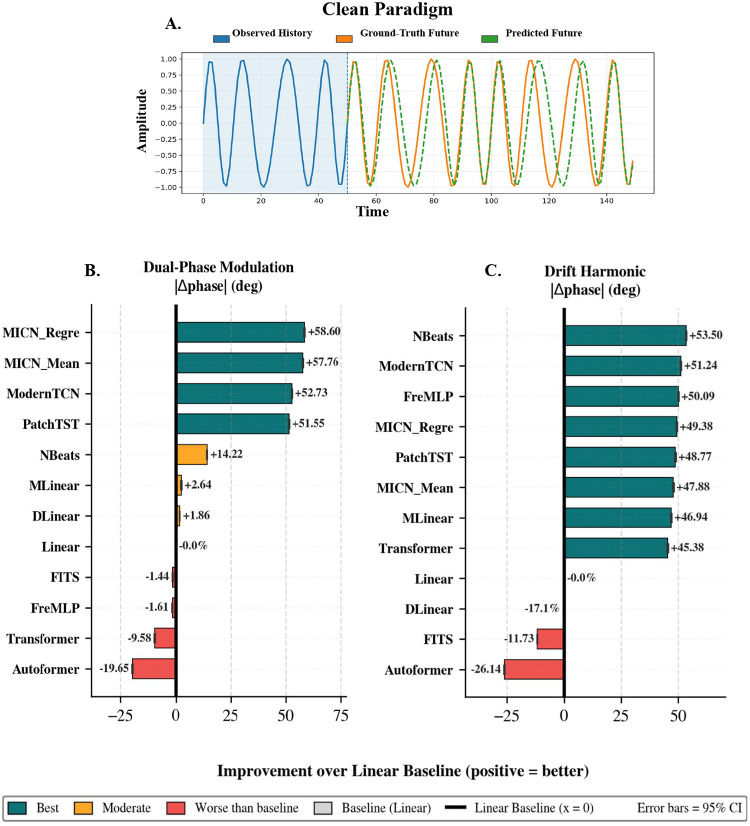
PatchTST, MICN, and ModernTCN achieve > 50° phase-accuracy improvement on dual-phase signals, but rankings reverse on simpler signals. (**a**) Clean paradigm: models observe history (blue) and predict the future (green) against ground truth (orange); peak misalignment is invisible to pointwise metrics. (**b**) Phase-accuracy improvement (∣Δphase∣) over the linear baseline on dual-phase signals. (**c**) The same on drift-harmonic signals, where the ranking reverses (NBeats and ModernTCN lead and Transformer exceeds baseline), indicating that broad attention preserves phase on simple spectra but fails under multi-frequency modulation. Error bars are 95% confidence intervals; tests are paired t-tests with Holm correction.

We began by asking which families preserve oscillatory structure with no perturbation present, a baseline that any reliable digital-twin candidate must pass. On the most demanding signals (dual-phase modulation), families separated sharply (Fig. 4a,b). MICN_Regre (+58.60°), MICN_Mean (+57.76°), ModernTCN [20] (+52.73°), and PatchTST (+51.55°) each improved phase accuracy by more than 50° over the linear baseline (all p<0.05), while Transformer (−9.58°) and Autoformer [24] (−19.65°) degraded below it, indicating that broad attention disrupts phase under multi-frequency modulation. FreMLP showed a small deficit $\left(-1.61^{{}^{\circ}},p<0.05\right)$ consistent with spectral leakage.

The ranking shifted systematically with complexity. On drift-harmonic signals, where a single slowly varying frequency dominates, nearly all architectures surpassed baseline, including Transformer (+45.38°), while FITS (−11.73°) and Autoformer (−26.14°) degraded. NBeats showed the strongest complexity dependence, rising from +14.22° on dual-phase to +43.72° on single-phase and +53.50° on drift-harmonic signals; FreMLP followed the same pattern (−1.61° to +50.09°). Autoformer remained the weakest architecture across all families and dimensions. In short, architectures processing the input through localized temporal windows (PatchTST, MICN, ModernTCN) preserved oscillatory structure even on complex signals, whereas global mappings and full-sequence attention held fidelity only when spectral structure was simple. This is the earliest instance of a pattern that recurs under every perturbation below.

### Localized architectures degrade gracefully under noise

**Fig. 5:**
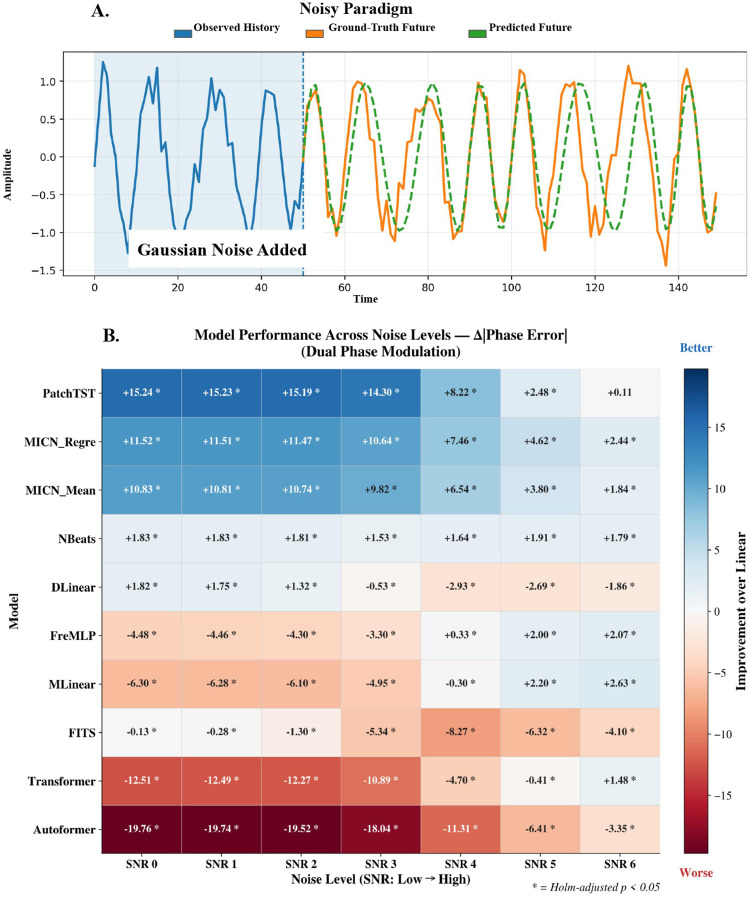
PatchTST and MICN variants maintain phase accuracy under noise; linear models degrade systematically. (**a**) Noisy paradigm: models trained on clean signals; at test time Gaussian noise is added to observed history (blue) while the future remains clean. (**b**) Phase-error difference across increasing noise (SNR 0 to SNR 6, higher = more severe) on dual-phase signals. Per-signal-family analyses are in Supplementary Figs. A4–A5. Differences marked * are Holm-corrected p<0.05.

Biosignals are invariably corrupted by motion artifact in PPG, impedance fluctuation in ECG, and muscle activity in EEG [5, 7]. Training on clean signals and testing on history corrupted with additive Gaussian noise across six SNR levels (clean future targets; Methods), the locality pattern persisted (Fig. 5). On dual-phase signals, PatchTST led at low-to-moderate corruption (+15.24° at SNR 0; +8.22° at SNR 4), while MICN_Regre and MICN_Mean retained significant improvement across the full range, including under severe corruption (SNR 6: +2.44° and +1.84°) where PatchTST’s advantage fell to non-significance (+0.11°). Autoformer showed the largest degradation at every level (−19.76° to −3.35°), and DLinear crossed from positive to negative as noise rose (+1.82° at SNR 0 to −1.86° at SNR 6).

A consistent division of labor emerged across families: PatchTST led under mild noise, while MICN’s multi-scale convolution gave more stable retention under severe corruption. On single-phase signals MICN_Regre held +26.37° at SNR 6, outperforming PatchTST by +8.91°. Autoformer remained the worst performer across all three families. The complementary robustness profiles of PatchTST and MICN suggest that architecture choice should be matched to the expected noise regime, or combined in an ensemble.

### Frequency-shift adaptation is architecture-dependent

**Fig. 6:**
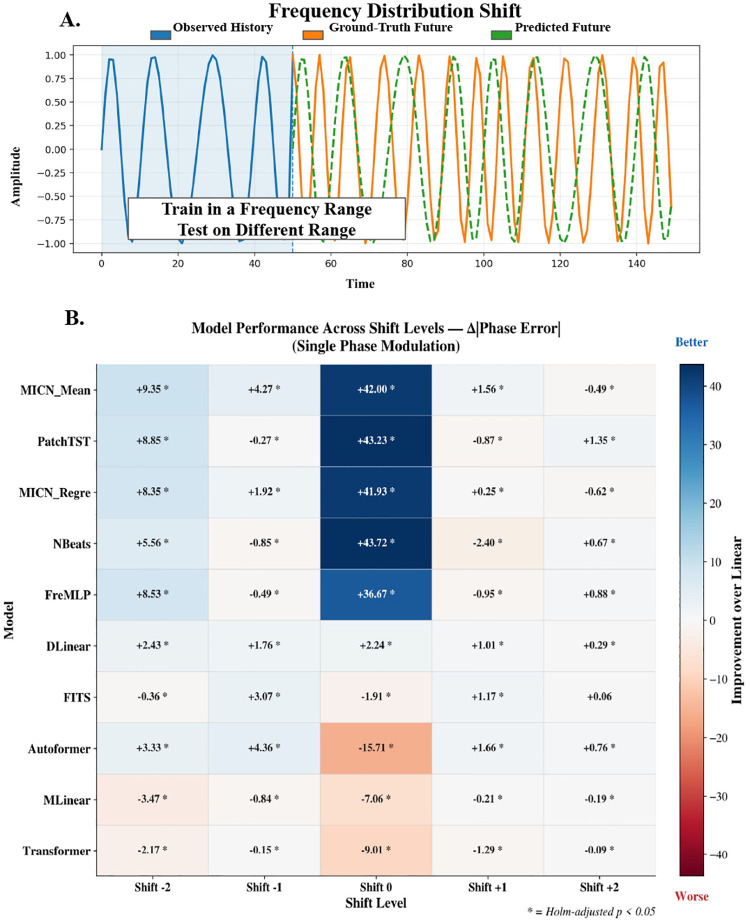
PatchTST and MICN variants adapt better to rhythm changes; linear and full-sequence attention models degrade across frequency shifts. (**a**) Frequency-shift condition: models trained on one frequency range (history, blue) forecast into a shifted range (future, orange/green). (**b**) Phase-error difference across shift levels (Shift −2 to +2) on single-phase signals. Per-signal-family analyses are in Supplementary Figs. A6–A7. Differences marked * are Holm-corrected p<0.05.

Physiological rhythms are nonstationary: heart rate rises with exercise, respiratory rate changes with posture, and EEG bands shift across arousal states [5, 7]. Testing models trained on one frequency band on distributions displaced by −2 to +2 range-widths (Methods), the localized architectures again led at moderate shift (Fig. 6). On single-phase signals, NBeats (+43.72°), PatchTST (+43.23°), MICN_Mean (+42.00°), and MICN_Regre (+41.93°) all exceeded +40° at no shift; under shift these declined gracefully rather than collapsing, with MICN_Mean (+9.35°) and PatchTST (+8.85°) retaining the most fidelity at Shift −2. By contrast, Transformer (−9.01°) and Autoformer (−15.71°) degraded below baseline even without any shift, indicating that global attention suppresses rather than tracks frequency-specific phase relationships.

Degradation scaled with complexity and showed informative asymmetries. On dual-phase signals the collapse was severe and roughly symmetric (MICN_Regre: +58.60° at Shift 0 to −4.57° at Shift −2); on drift-harmonic signals it was directional, with most architectures degrading more under downward than upward shifts (MICN_Mean: −10.32° at Shift −2 versus −1.94° at Shift +2), implying downward shifts are harder for models trained on higher-frequency bands. No architecture retained more than approximately 10° at extreme shifts (±2), marking a boundary on out-of-distribution generalization; within that boundary, PatchTST and MICN provided the strongest adaptation where linear and attention models had already failed.

### PatchTST and ModernTCN adapt fastest to observable state transitions

The most clinically consequential events, including atrial-fibrillation onset, the interictal-to-ictal transition, and sleep-stage shifts [7], raise a question no deterministic forecaster can sidestep: not whether it can predict an unseen transition, but how quickly it adapts once evidence of the new state enters the observable history. We introduced deterministic frequency transitions at varying distances from the forecast boundary and tracked phase recovery as post-transition context accrued (in-context tags H2–H40; Methods).

**Fig. 7:**
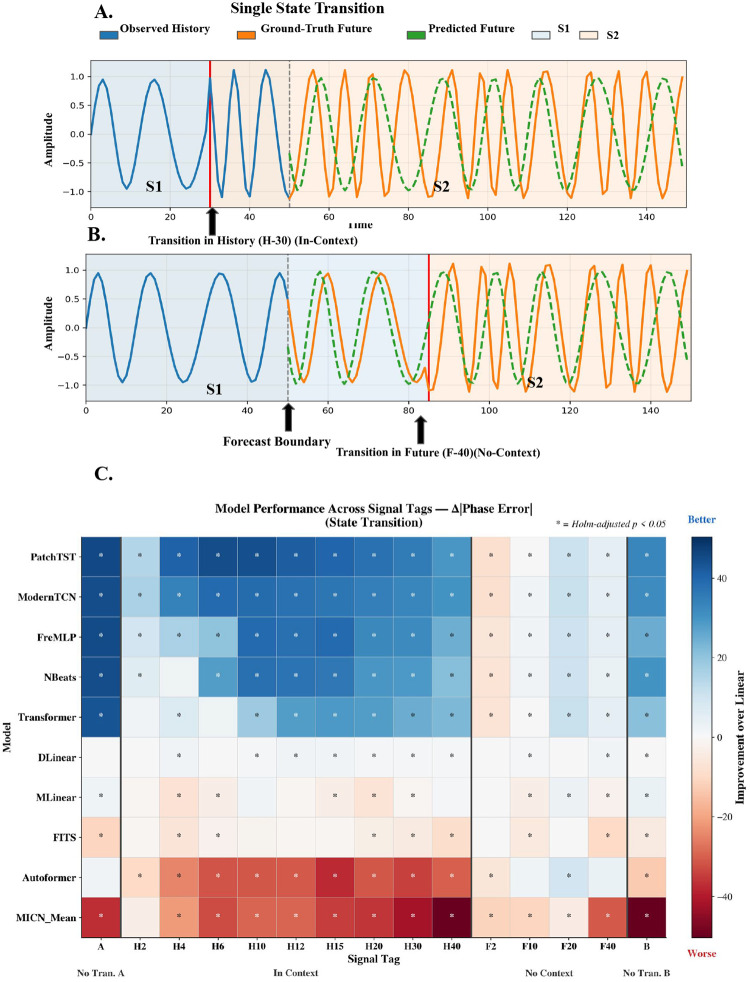
PatchTST and ModernTCN adapt fastest to observable state transitions. **(a)** Single S1→S2 transition within the observed history (H-30, in-context), providing post-transition context before the forecast boundary. (**b**) Transition in the unobserved future (F-40, no-context). (**c**) Phase improvement over the linear baseline versus post-transition context: in-context (H2–H40) and no-context (F2–F40), where all models forecast the pre-transition state. Per-dimension analyses are in Supplementary Figs. A8–A9. Differences marked * are Holm-corrected p<0.05.

Architectures processing short, overlapping windows adapted fastest (Fig. 7). PatchTST and ModernTCN recovered to within 20° of their no-transition baselines (4.7° and 5.8°) within just 6 timesteps of context (both p<0.05 versus Linear at H6). FreMLP and NBeats needed approximately 15 timesteps; Transformer required approximately 40, reaching 15.4° only at H40, with global attention diffusing transition evidence across the full context rather than concentrating it at the change point. When transitions fell in the unobserved future (F2–F40), all models forecast the pre-transition state, confirming the paradigm tests adaptation to evidence, not clairvoyance. The hierarchy held beyond phase: PatchTST and ModernTCN recovered fastest across amplitude and frequency too, whereas MICN_Mean dissociated, with strong frequency adaptation (p<0.05 at H10) but persistent amplitude degradation (H6 through H40), recovering rhythm before magnitude and illustrating why separate diagnostics are necessary. At 10 Hz sampling, PatchTST’s 6-timestep recovery corresponds to approximately 0.6 s of context versus approximately 4 s for Transformer, a concrete measure of adaptation lag. Notably, the MICN variants that excelled under noise and shift did not lead here, the only paradigm to separate the localized architectures from one another.

### No architecture reliably recovers stochastic switching dynamics

Finally, cardiac rhythm, sleep staging, and autonomic tone fluctuate stochastically [5, 6], and conditions such as paroxysmal atrial fibrillation are defined by switching statistics rather than any single waveform [7]. We generated signals from a two-state Markov chain (p∈{0.10,0.30,0.50,0.70,0.90}) and asked whether each forecast preserved the switching statistics of the ground-truth future, scored by symmetric KL divergence between switching-probability distributions recovered by a standardized two-state Gaussian HMM probe applied identically to true and predicted signals (recovery summarized at symmetric KL < 0.05; Methods).

This paradigm exposed a limitation no architecture overcame. PatchTST was the only model to recover switching at more than one transition probability (p=0.70,KL=0.008;p=0.90,KL=0.046), both at high probabilities where frequent alternation supplies more within-window evidence. Six architectures recovered at a single probability each (DLinear, Linear, FreMLP, ModernTCN, MICN_Mean, MICN_Regre; KL = 0.014–0.034), and five failed everywhere (Autoformer, FITS, MLinear, NBeats, Transformer; KL up to 2.020). NBeats’ complete failure is striking given its fast deterministic-transition adaptation, showing that adapting to an observable transition and preserving probabilistic switching are distinct capabilities. Even PatchTST succeeded in fewer than half the conditions. The continuous KL values and a threshold-sensitivity analysis preserve this ranking, and the uniform difficulty across all four families indicates the limitation is architectural-general: deterministic input-output forecasters are suited to waveform extrapolation, not to reproducing transition structure.

### Localized temporal processing achieves the most balanced fidelity

Each preceding test isolates one perturbation, but a deployed digital twin faces them jointly. To identify architectures that hold fidelity across all five paradigms simultaneously, we performed a Pareto analysis, training each model separately per paradigm to isolate architectural effects (Methods). Four models reached the frontier, but for different reasons: MICN_Regre and MICN_Mean through clean, noise, and shift performance but weak state transitions (normalized 0.54 and 0.00); ModernTCN through state transitions alone (0.97) at the cost of noise (0.31) and shift (0.23). Only PatchTST exceeded 0.8 on every dimension (clean 0.97, noise 0.98, shift 1.00, transition 1.00, Markov 1.00); NBeats, though dominated, placed third overall (0.85 mean, nothing below 0.77). No other architecture exceeded 0.8 on more than three dimensions.

The same answer thus emerged under every perturbation: localized temporal processing preserved physiological fidelity, global mappings and full-sequence attention did not, and no deterministic architecture captured stochastic switching. Among all models, PatchTST held the most balanced fidelity across noise, shift, deterministic transitions, and switching. For health-signal digital twins, architectures with localized temporal processing are therefore the strongest candidates for further development, though final model choice should be matched to the perturbation dimensions most relevant to the target application and validated on real patient recordings.

## 3 Discussion

Using TimeSynth, we found that conventional accuracy metrics obscure how forecasting architectures preserve the oscillatory, nonstationary, and stochastic dynamics of physiological signals, and that architectural choice strongly determines what a digital twin forecast can faithfully represent. This speaks directly to a recognized gap: the NASEM report identifies verification, validation, and uncertainty quantification as prerequisites for trustworthy digital twins [4], yet such frameworks for biosignal forecasting remain underdeveloped [11, 12]. Synthetic signals with analytically known dynamics are what make this verification possible: ground-truth amplitude, frequency, and phase trajectories cannot be recovered from real recordings, so controlled generation is a prerequisite for the stress test rather than a weakness of it, the same enabling role that synthetic data plays in other physiological deep-learning systems [15]. TimeSynth accordingly provides a controlled preclinical environment for exposing dynamical failure modes before forecasting models are coupled to real patient time series.

The central finding is a dissociation between pointwise accuracy and dynamical fidelity (Fig. 3): models with comparable MAE differed by up to 53° in phase error, so a forecast can appear accurate by standard benchmarks while mistracking oscillatory timing. The clinical scale of such an error depends on signal frequency. A 53° phase error corresponds to roughly 123 ms for a 1.2 Hz cardiac rhythm, a magnitude relevant to R-R interval variability and rhythm monitoring [5]; to 12–18 ms in the EEG alpha band (8–12 Hz), where it may disrupt phase-sensitive neural analyses [7]; and to nearly 590 ms for respiratory-modulated PPG near 0.25 Hz, potentially obscuring respiratory-cardiovascular coupling [7]. These translations do not demonstrate clinical failure, but they identify timing errors invisible to aggregate accuracy that warrant evaluation before deployment, consistent with concerns that many digital twin studies validate only on aggregate measures [11].

Across paradigms, the dominant determinant of fidelity was the locality of temporal processing. Patch-based and convolutional architectures preserved phase and frequency structure most consistently under clean, noisy, and shifted conditions (Figs. 4–6), and PatchTST achieved the most balanced fidelity across all five paradigms (Fig. 9), consistent with patch tokenization capturing local periodicity more effectively than pointwise mappings or broad-context attention [18] and with prior reports that vanilla Transformers underperform on structured time series [8]. The same mechanism accounts for adaptation speed after observable state changes: PatchTST and ModernTCN recovered to within 20° of baseline within six timesteps (about 0.6 s at 10 Hz), whereas full-sequence attention required roughly 40, diluting transition evidence across the input (Fig. 7). Locality was not sufficient on its own, however. MICN variants were strong under noise and shift but weak under deterministic transitions, and NBeats adapted to deterministic transitions yet failed to preserve stochastic switching, so no single inductive bias guaranteed fidelity across all regimes. The frequency-shift results marked a shared ceiling: no architecture retained more than about 10° of improvement at extreme shifts (±2 range-widths), with a directional asymmetry suggesting that rhythms moving away from the training band are intrinsically harder to track.

**Fig. 8:**
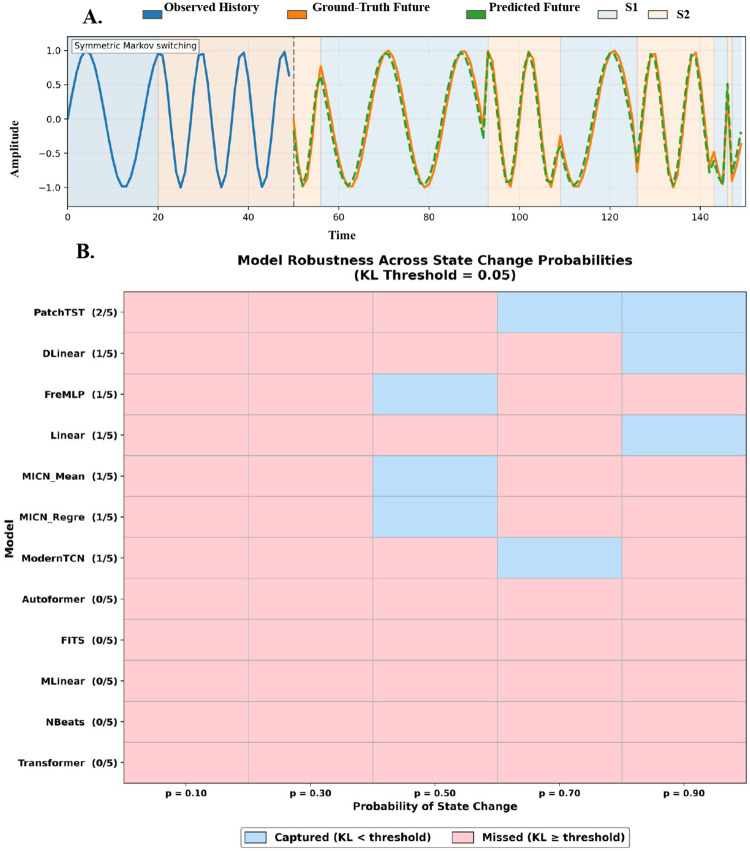
PatchTST partially recovers switching dynamics; most architectures show limited recovery. (**a**) Markov-switching signal alternating between states S1 and S2 with transition probability p. (**b**) Pass/fail summary across p=0.10 to 0.90 using the HMM probe; blue denotes recovery (symmetric KL < 0.05), red limited recovery. The HMM is a standardized downstream probe of switching statistics, not the true latent simulator. Full continuous KL values are in Supplementary Table A10; threshold-sensitivity analysis in Supplementary Fig. A10.

**Fig. 9:**
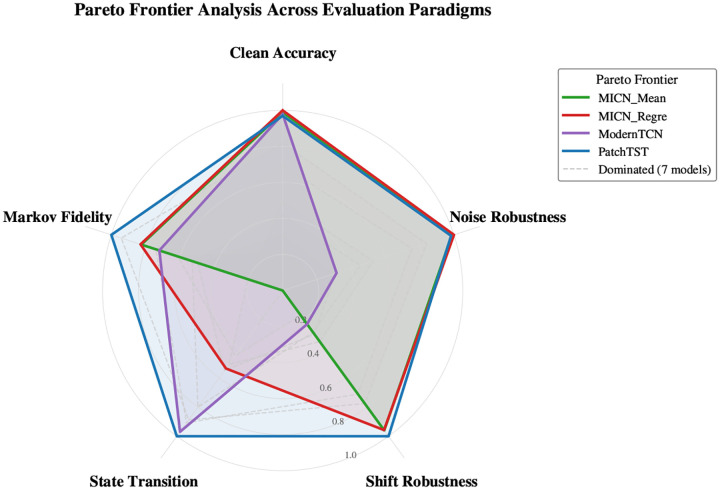
PatchTST maintains the most balanced fidelity across all evaluation paradigms. Radar plot of 11 models across five paradigms (clean accuracy, noise robustness, shift robustness, state transition, Markov fidelity), each normalized to 0–1. Solid polygons denote Pareto-optimal models; dashed gray polygons denote dominated models. Axes represent aggregate improvement over the linear baseline, averaged across signals and conditions. Full normalized scores are in Supplementary Table A11.

The sharpest limitation appeared under stochastic switching (Fig. 8), where most architectures failed and even PatchTST recovered the switching statistics in only two of five regimes. We assessed recovery with a two-state Gaussian HMM applied identically to true and predicted signals as a standardized downstream probe, not as an oracle or as the true latent simulator, asking whether a forecast preserves enough temporal-spectral structure for comparable switching statistics to be inferred; the conclusion held across continuous KL values and a threshold-sensitivity analysis. Interpreted this way, the failure is informative: deterministic point forecasters can reproduce plausible waveforms while discarding distributional state-transition structure. Forecasting models for digital twins may therefore require explicit latent-state, probabilistic, or distributional mechanisms rather than point-estimate prediction alone.

These results reframe how forecasting models for digital twins should be selected and validated. Because no single architecture dominates every fidelity dimension, the practical conclusion is not a universal winner but a principled selection rule, mirroring how benchmarking of synthetic-data generators in other biomedical domains resolves into use-case-specific guidance rather than a single ranking [17]. Selection should therefore weigh balanced fidelity across perturbations rather than clean-condition accuracy alone, with the weighting matched to the use case: ambulatory cardiac monitoring may prioritize noise robustness, intensive care may prioritize rapid adaptation to state changes, and sleep or seizure applications may prioritize preservation of switching statistics. Beyond benchmarking, TimeSynth offers two further roles for model development. Its signals with known ground-truth dynamics can support physiologically structured pretraining before fine-tuning on limited patient data, consistent with evidence that well-designed synthetic data can rival domain-specific training [25], and its fidelity diagnostics provide interpretable feedback on which dynamical properties an architecture preserves or destroys. A natural extension is to move from analytic generators to constrained generative models trained on real biosignals, with latent variables aligned to amplitude, frequency, phase, and state transitions, increasing realism while retaining the interpretability that makes the framework useful for verification.

Several limitations qualify these conclusions. The benchmark uses univariate signals, fixed input-output horizons, and deliberately simplified perturbations, whereas clinical digital twins typically require multivariate inputs, irregular sampling, patient-specific adaptation, and uncertainty quantification. Physiological dynamics also vary with age, sex, comorbidity, sensor type, and care setting [13], so architectures intended for broad use should be evaluated across patient subgroups to ensure that fidelity failures are not unevenly distributed. Finally, the switching analysis depends on a downstream HMM probe and should not be read as definitive latent-state validation; comparing alternative probes and distributional distances on real state-transition datasets is an important next step.

In summary, pointwise accuracy is insufficient for evaluating forecasting models intended for health-signal digital twins. Architectures with localized temporal processing, particularly PatchTST, MICN, and ModernTCN, better preserved amplitude, frequency, and phase fidelity under controlled perturbations, while deterministic architectures generally struggled with stochastic switching. TimeSynth provides a controlled preclinical framework for exposing these failure modes, guiding architecture development, and clarifying which models warrant further evaluation in patient-specific digital twin workflows.

## 4 Methods

### Synthetic signal generation

We generated synthetic signals from closed-form parametric models whose terms map to interpretable physiological quantities, in three families each grounded in a real biosignal modality. Single phase-modulated (SPM) signals model quasi-periodic dynamics with one source of phase variation, using a carrier whose phase is sinusoidally modulated at a slower rate:

(1) $$x(t)=A\;\text{sin}\left(2\pi ft+\beta \;\text{sin}\left(2\pi f_{\text{mod}}t\right)\right)+c,$$

where A is the carrier amplitude, f the carrier frequency (Hz), β the phase-modulation index, $f_{\text{mod}}$ the modulation frequency (Hz), and c a DC offset. Dual phase-modulated (DPM) signals sum two independently modulated components with a shared offset, capturing multi-component harmonic structure:

(2) $$x\left(t\right)=\sum_{i=0}^{1}A_{i}\;\text{sin}\left(2\pi f_{i}t+\beta_{i}\;\text{sin}\left(2\pi f_{\text{mod},i}t\right)\right)+c,$$

with each component’s parameters $\left(A_{i},f_{i},\beta_{i},f_{\text{mod},i}\right)$ sampled independently from the SPM bounds. Drift-harmonic (DH) signals combine a carrier with a time-varying amplitude envelope and linear trend, reproducing slow nonstationarity in peripheral cardiovascular signals:

(3) $$x(t)=\left(1+\varepsilon \;t\right)\text{sin}(2\pi ft+\varphi )+at,$$

where ε is the envelope drift rate (fixed at −0.05 across realizations), φ the initial phase, and a the linear trend coefficient; DH signals were min-max normalized to [0, 1].

To ground parameter ranges in physiology, we fit each model to real segments from three public datasets: the MIT-BIH Arrhythmia Database [22] (ECG, 48 records, 360 Hz), PPG-DaLiA [21] (PPG/BVP, 15 subjects, 64 Hz), and the CHB-MIT Scalp EEG Database [23] (EEG, channel FP1-F7). SPM and DPM were fit to the smooth oscillatory S-Q-interval baseline of MIT-BIH ECG, DH to PPG-DaLiA blood-volume-pulse segments, and the extended DPM model to CHB-MIT EEG. Each model was implemented as a differentiable parametric module in PyTorch and optimized with Adam over overlapping sliding windows, with physiological plausibility enforced by per-step box-constraint clamping on learnable parameters. The fitted ranges for carrier frequency, modulation depth, amplitude, phase, offset, and drift rate defined the generation space for each family (Fig. 2; Supplementary Tables A1, A2).

Signals were sampled at $f_{s}=10\;\text{Hz}$ for 300 s (3,000 points per instance). The 10 Hz rate captures modulation-envelope dynamics relevant to digital twin forecasting rather than high-frequency waveform morphology, since cardiac, respiratory, and neural monitoring often operate on derived quantities such as heart rate, respiratory rate, and spectral power that evolve over seconds to minutes. Each family contained 100 unique parametric realizations split into 70 training, 10 validation, and 20 test instances, with uniqueness enforced by deterministic parameter hashing (Supplementary Appendix A1.3).

### Controlled evaluation paradigms

We evaluated forecasters under five conditions that stress-test dynamical fidelity in settings relevant to health-signal digital twins. In the **clean** paradigm, models train and test on noise-free signals, establishing a per-family ceiling for each fidelity dimension. For **noise robustness**, models trained on clean signals are tested across seven SNR levels, a clean baseline (SNR 0) plus six additive-Gaussian-noise levels from 40 to 1 dB (SNR 1 to SNR 6, higher index more severe), with noise power calibrated relative to zero-mean signal power so that the DC offset does not inflate the estimate (Supplementary Appendix A2.1). For **frequency distribution shift**, models trained on one frequency range are tested on bands displaced by −2 to +2 range-widths; within each shifted band, 20 test signals are generated with carrier frequency sampled uniformly from the shifted range and all non-frequency parameters held at training bounds (Supplementary Appendix A2.2; Supplementary Table A4). For the **single state transition** paradigm, a deterministic frequency change-point is placed either within the observed history (in-context, tags H2–H40) or in the unobserved future (no-context, tags F2–F40), with phase continuity maintained by recursive accumulation to avoid artificial discontinuities at the change-point; each split contains 600 training, 100 validation, and 200 test instances (Supplementary Appendix A2.3). For **Markov switching**, signals alternate stochastically between two frequency states under a symmetric two-state Markov chain with transition probabilities p∈{0.10,0.30,0.50,0.70,0.90}, with 70 training, 10 validation, and 20 test instances per value of p (Supplementary Appendix A2.4). Together these paradigms approximate sensor noise, evolving rhythms, abrupt state changes, and stochastic variability encountered in patient monitoring; full specifications are provided in Supplementary Appendix A2.

### Forecasting models and inductive-bias hypotheses

We benchmarked 11 models spanning four architectural families selected to represent dominant approaches in time series forecasting and to isolate how temporal processing affects dynamical fidelity. The **linear** family (Linear, DLinear [8], FITS [26]) maps input to output through learned linear projections. The **MLP** family (MLinear, NBeats [27], FreMLP [28]) adds nonlinear transformations, with FreMLP operating in the frequency domain. The **convolutional** family (ModernTCN [20], MICN [19]) processes inputs through convolutional kernels over local windows, with MICN evaluated in two trend-prediction variants (MICN_Mean, MICN_Regre). The **transformer** family (Transformer, Autoformer [24], PatchTST [18]) uses attention ranging from full-sequence to patch-based.

Our analysis groups these models by the inductive bias most relevant to physiological dynamics, namely the locality of temporal processing. Linear and full-sequence attention models (Transformer, Autoformer) integrate information globally, whereas convolutional (ModernTCN, MICN) and patch-based (PatchTST) models process short local windows; although PatchTST is attention-based, its patchwise tokenization first constrains the model to local temporal segments, giving it an effectively localized bias. We therefore hypothesized that localized processing would better preserve phase, short-range frequency structure, and recent state evidence, so that convolutional and patch-based models would retain fidelity under clean conditions, degrade more gracefully under noise, adapt faster once post-transition context becomes observable, and remain more robust under moderate frequency shifts, whereas linear and full-sequence attention models would preserve fidelity only when spectral structure is simple. We further expected stochastic Markov switching to be the hardest paradigm for all deterministic forecasters, since it requires preserving transition probabilities and dwell-time structure rather than extrapolating a single most-likely waveform.

All models were configured for univariate forecasting with a 50-step input and a 100-step prediction horizon. The 50-step input (5 s at 10 Hz) spans roughly five oscillatory cycles at a 1 Hz cardiac frequency, sufficient to observe the carrier and its modulation envelope; the 100-step horizon (10 s) tests whether fidelity is sustained over an extended window. Models were trained under a unified protocol (AdamW optimizer, OneCycleLR scheduling, MSE loss, batch size 128, early stopping on validation loss), independently on each signal type and paradigm to isolate architectural effects from task-specific optimization. Architectural descriptions, model-specific hyperparameters, and family-level deviations are reported in Supplementary Appendix A3.

### Fidelity metrics

We evaluate forecasts along three complementary dimensions that isolate distinct failure modes: whether the prediction preserves oscillation magnitude, rate, and timing. Standard pointwise metrics conflate these and can mask a failure in one dimension behind acceptable performance in another. Amplitude error is the mean absolute error (MAE) over the H-step horizon,

(4) $$\text{MAE}=\frac{1}{H}\sum_{t=1}^{H}|\overset{ˆ}{y}(t)-y(t)|,$$

capturing oscillation magnitude, the property most directly measured by conventional benchmarks. Frequency error captures mismatch in dominant oscillation rate, computed from the power spectrum $P(k)=|ℱ(x){|}^{2}$ after DC removal. The peak bin $k^{*}$ is refined by three-point parabolic interpolation,

(5) $$\overset{ˆ}{f}=\left(k^{*}+\frac{P\left(k^{*}-1\right)-P\left(k^{*}+1\right)}{2\left(P\left(k^{*}-1\right)-2P\left(k^{*}\right)+P\left(k^{*}+1\right)\right)}\right)\cdot \frac{f_{s}}{N},$$

where N is the FFT length; estimates with peak power below 10% of total spectral power are discarded, and frequency error is $\left|{\overset{ˆ}{f}}_{\text{pred}}-{\overset{ˆ}{f}}_{\text{true}}\right|$. Phase error captures temporal misalignment via the analytic signal z(t), obtained by the frequency-domain Hilbert transform (zeroing negative-frequency bins, doubling positive-frequency bins, inverse-transforming with zero-padding at pad factor 2 to limit edge effects). Instantaneous phase is φ(t)=unwrap(arg(z(t))), and to avoid spurious estimates in low-amplitude regions the difference is evaluated only where the true instantaneous amplitude exceeds 20% of its median,

(6) $$\Delta \varphi =\frac{1}{\left|ℳ\right|}\sum_{t\in ℳ}\left|{\text{wrap}}_{\pi }\left(\varphi_{\text{pred}}(t)-\varphi_{\text{true}}(t)\right)\right|,\;ℳ=\left\{t:\left|z_{\text{true}}(t)\right|>0.2\cdot \text{median}\left(\left|z_{\text{true}}\right|\right)\right\},$$

where ${\text{wrap}}_{\pi }$ maps the difference to (-π,π] and phase error is reported in degrees. A model may achieve low MAE while mistracking oscillation rate or peak timing, so only these separate diagnostics distinguish such failure modes. Full implementation details, including padding factors, smoothing windows, and edge-case handling, are provided in Supplementary Appendix A4.

### Statistical analysis

Models were compared using paired tests on per-sequence metric distributions, ensuring each model was evaluated on the same sequences. For frequency and phase error, where reliability filtering can produce missing values, intersection-valid masking included a sequence only if all compared models had finite values for that metric, preventing differences in sample composition from confounding the comparison. For each metric we computed paired differences against the Linear baseline per sequence. For the clean, noise, and frequency-shift paradigms, significance was assessed with paired t-tests and 95% confidence intervals $\left(\overset{‾}{d}\pm 1.96s_{d}/\sqrt{n}\right)$; for state-transition analyses, where mixed in-context and no-context conditions yield non-Gaussian distributions, we used the Wilcoxon signed-rank test with tie correction. Within each metric and paradigm, p-values were adjusted for multiple comparisons using the Holm step-down procedure, controlling the family-wise error rate at α=0.05 with greater power than Bonferroni correction.

To assess whether forecasts preserve stochastic switching statistics, we used a two-state Gaussian hidden Markov model (HMM) as a standardized downstream probe rather than as the true latent model. The HMM was fit to windowed spectral features (Welch periodogram, window 16, hop 8) of the true history, selecting the best of eight random seeds by log-likelihood, then applied unchanged to true and predicted futures. Decoded switching-probability distributions were compared using symmetric KL divergence, which penalizes both under- and over-switching. The KL_sym_ < 0.05 cutoff was used only for heatmap summarization; continuous values and threshold sensitivity at 0.05, 0.10, 0.15, and 0.20 are reported in Supplementary Fig. A10 and Supplementary Appendix A5.5.

To identify architectures that maintain fidelity across all conditions, we performed a Pareto analysis over the five paradigms (clean accuracy, noise robustness, shift robustness, state-transition adaptation, and Markov fidelity). Within each paradigm, scores were computed as aggregate improvement over the Linear baseline and min-max normalized to [0, 1] across models. A model was classified as Pareto-optimal if no other model matched or exceeded it on all five dimensions while strictly exceeding it on at least one.

## Supplementary Material

1

## Figures and Tables

**Table 1:** Physiological events and the synthetic signals used to simulate them in TimeSynth.

Physiological event	Synthetic signal & perturbation
Baseline wander (ECG, EEG)	Drift-harmonic (low-frequency drift)
Respiration coupling (PPG)	Single phase modulation
Heart rate variability (ECG)	Dual phase modulation + Markov switching
Neural oscillation variability (EEG)	Multi-frequency modulation + state switching
Sensor noise (all signals)	Additive noise (SNR 0–6 dB)
Exercise-induced rhythm change	Non-stationary frequency shift
Arrhythmia onset (ECG)	Single deterministic state transition
Sleep-stage transition (EEG)	State transition + Markov switching
Electrode artifact (ECG, EEG)	Drift + additive noise

## Data Availability

The three source datasets are publicly available: the MIT-BIH Arrhythmia Database and CHB-MIT Scalp EEG Database via PhysioNet, and PPG-DaLiA via the UCI Machine Learning Repository. The synthetic signal families generated for this study, together with the fitted parameter distributions used to produce them, can be regenerated exactly using the released code (https://github.com/RakibulHaqueSajal/TimeSynth), so that all evaluation paradigms are fully reproducible.

## Code availability

TimeSynth is released as reusable infrastructure at https://github.com/RakibulHaqueSajal/TimeSynth: the physiologically grounded signal generator, the controlled perturbation paradigms, the amplitude, frequency, phase, and state-transition fidelity diagnostics, and the scripts reproducing every figure and statistical test. The release is intended to let others benchmark new architectures, generate controlled ground-truth signals for pretraining, and audit fidelity in their own digital-twin workflows.
