# Supplementary material for: Timesynth: A Temporal Fidelity Framework for Health Signal Digital Twins
Source: Res Sq. 2026 Jul 1:rs.3.rs-10144018. Preprint. [Version 1] doi: 10.21203/rs.3.rs-10144018/v1 (PMC13345539; doi:10.21203/rs.3.rs-10144018/v1)
Supplement: 1 [file NIHPPRS10144018V1-supplement-1.pdf]

Appendix A: Supplementary Information

This appendix provides the full technical specifications, supporting analyses, and extended results for the TimeSynth framework. It is organized as follows: **A1** synthetic signal generation, **A2** controlled evaluation paradigms, **A3** forecasting model architectures and hyperparameters, **A4** fidelity metric computation, **A5** statistical analysis, and **A6** extended results across signal families and evaluation paradigms.

A1 Synthetic Signal Generation

A1.1 Extended signal model for EEG fitting

The SPM, DPM, and DH signal family equations are defined in the main text (Eqs. 1–3). For fitting to real EEG recordings, the DH model was extended with additional frequency bands and transient Gaussian spike components to accommodate the multi-band structure and transient deflections characteristic of scalp EEG:

$$x(t) = \sum_{i=1}^{n_{\text{bands}}} A_i\big(1+e_i \sin(0.5\pi t)\big)\sin(2\pi f_i\, t+\varphi_i)+\sum_{j=1}^{n_{\text{spikes}}} A_j^{(\text{sp})}\exp\Big(-\frac{(t-t_{c,j})^2}{2\sigma_j^2}\Big) \quad \text{(A1)}$$

where $n_{\text{bands}} = 2$ frequency bands and $n_{\text{spikes}} = 2$ transient Gaussian spike components, e_i denotes the envelope modulation depth, $A_j^{(\text{sp})}$ and $t_{c,j}$ the spike amplitude and center, and σ_j the spike width. This richer model was used exclusively for parametric fitting to CHB-MIT recordings to derive physiologically grounded parameter bounds; the synthetic DH signals used for evaluation were generated from the simpler closed-form equation defined in the main text (Eq. 3).

A1.2 Parametric fitting to real biosignals

To derive physiologically grounded parameter bounds, each parametric model was fit to real signal segments from three clinical-grade datasets. Fitting was performed using differentiable parametric modules implemented in PyTorch and optimized with Adam. After each gradient step, all learnable parameters were clamped to predefined physiological bounds (box constraints). Fitting hyperparameters for each dataset are summarized in Table A1.

PPG fitting.

Blood volume pulse (BVP) recordings from PPG-DaLiA (15 subjects, $f_s = 64$ Hz) were fit using the drift-harmonic model (main text Eq. 3) with learnable parameters ε, f, φ, and a. Segments of 5.0 s with 50% overlap were optimized for 500 epochs using MSE loss (lr = 0.01). Parameter bounds: $\varepsilon \in [-0.05, 0.05]$, $f \in [0.85, 1.1]$ Hz, $\varphi \in [-\pi/4, \pi/4]$ rad, $a \in [-0.1, 0.1]$.

ECG fitting.

S-Q interval segments from the MIT-BIH Arrhythmia Database (48 records, f_s = 360 Hz, 10 s per record) were fit using the phase-modulated multisine model (main text

Eq. 2) with $N = 2$ components. Segments of 1.0 s with 15% overlap and 10% blend ratio were optimized for 500 epochs using L1 loss (lr = 0.001). Parameter bounds: $A_i \in [0.1, 0.4]$, $f_i \in [0.5, 3.0]$ Hz, $\beta_i \in [0.01, 0.3]$, $f_{\mathrm{mod},i} \in [0.01, 0.1]$ Hz, $c \in [0.0, 1.0]$.

EEG fitting.

Scalp EEG recordings from the CHB-MIT database (channel FP1-F7, 100 s extracted segments) were fit using the extended multi-band model (Eq. A1) with $n_{\mathrm{bands}} = 2$ and $n_{\mathrm{spikes}} = 2$. Signals were normalized to $[-1, 1]$ prior to fitting. Segments of 1.0 s were optimized for 100 epochs using L1 loss (lr = 0.01).

Cosine blending.

Overlapping fitted segments were combined using cosine ramp weights to prevent discontinuities at segment boundaries:

$$w(n) = \frac{1}{2}\Big(1 - \cos\big(\tfrac{\pi n}{N-1}\big)\Big), \quad n = 0, 1, \ldots, N-1 \tag{A2}$$

where N is the segment length. The final fitted signal at each time point is the weighted average $\hat{x}(t) = \sum_k w_k(t)\hat{x}_k(t) \big/ \sum_k w_k(t)$, summing over all segments k covering time t.

Table A1 Parameter bounds for synthetic signal generation. All parameters are sampled from uniform distributions over the stated ranges unless marked as fixed.

Signal family	Parameter	Symbol	Range	Unit
SPM	Carrier amplitude	A	[0.1, 0.1227]	–
SPM	Carrier frequency	f	[0.6782, 1.4112]	Hz
SPM	Modulation index	β	[0.01, 0.3]	rad
SPM	Modulation frequency	f_{mod}	[0.01, 0.1]	Hz
SPM	DC offset	c	[0.1937, 0.7418]	–
DPM	Per-component parameters	$A_i, f_i, \beta_i, f_{\mathrm{mod},i}$	Same as SPM	–
DPM	Shared offset	c	[0.1937, 0.7418]	–
DH	Envelope drift rate	ε	-0.05 (fixed)	s^{-1}
DH	Carrier frequency	f	[0.85, 1.10]	Hz
DH	Initial phase	φ	$[-0.65, 0.75]$	rad
DH	Linear trend coefficient	a	$[-6 \times 10^{-5}, 8 \times 10^{-5}]$	s^{-1}

A1.3 Generation procedure and parameter uniqueness

For each signal family, 100 unique parameter realizations were generated (70 training, 10 validation, 20 test). All parameters were sampled independently from uniform distributions over the bounds in Table A1 using a fixed random seed (seed = 42) for reproducibility.

Table A2 Fitting hyperparameters for real biosignal datasets. Seg. = segment length; Ovl. = overlap.

Dataset	Signal	f_s (Hz)	Seg. (s)	Ovl.	Epochs	lr	Loss	Model
PPG-DaLiA	PPG/BVP	64	5.0	50%	500	0.01	MSE	DH
MIT-BIH	ECG (S-Q)	360	1.0	15%	500	0.001	L1	SPM and DPM
CHB-MIT	EEG (FP1-F7)	–	1.0	–	100	0.01	L1	DH and spike

To guarantee that no two signals share the same parameter configuration, including across splits, each candidate parameter tuple was converted to a canonical string at six-decimal precision and hashed via MD5:

$$h = \mathrm{MD5}\big(\texttt{"}p_1\texttt{"}\_p_2\texttt{"}\_\cdots\texttt{"}\_p_k\texttt{"}\big) \tag{A3}$$

If the resulting hash matched any previously accepted configuration, the candidate was rejected and a new tuple was sampled. For SPM signals, the hash key comprised five values (A, f, β, f_{mod}, c). For DPM signals, it was extended to nine values (A_0, A_1, f_0, f_1, β_0, β_1, $f_{\mathrm{mod},0}$, $f_{\mathrm{mod},1}$, c). For DH signals, it covered f, φ, and a (with ε fixed).

All generation parameters were embedded directly in each output filename for full traceability. Signals were sampled at $f_s = 10$ Hz for 300 s, yielding $T = 3{,}000$ samples per instance over an evenly spaced time vector $t \in [0, 300)$.

The 10 Hz sampling rate was chosen to capture modulation-envelope dynamics relevant to digital twin operation rather than high-frequency waveform morphology. Clinical digital twins for cardiac, respiratory, and neural monitoring typically operate on derived features (heart rate, respiratory rate, spectral power) that evolve on timescales of seconds to minutes, and the modulation-envelope structure captured at 10 Hz represents the dynamical layer at which forecasting fidelity most directly impacts clinical decision-making.

A2 Controlled Evaluation Paradigms

The main text describes five evaluation paradigms in terms of their clinical motivation and what each tests for digital twin deployment. This section provides the full technical specifications, including signal generation equations, parameter separation protocols, phase-continuity mechanisms, and dataset sizes needed for reproduction.

A2.1 Noise robustness

Models trained exclusively on clean signals are evaluated at seven SNR levels (Table A3): a clean baseline (SNR 0) and six additive white Gaussian noise (AWGN) levels (SNR 1–6). Noise power is calibrated relative to the zero-mean signal power to

prevent the DC offset from inflating the power estimate:

$$P_{\text{sig}} = \frac{1}{T}\sum_{t=1}^{T}\big(x(t) - \bar{x}\big)^2 + \epsilon \tag{A4}$$

$$\sigma_{\text{noise}} = \sqrt{\frac{P_{\text{sig}}}{10^{\,\text{SNR}_{\text{dB}}/10}}} \tag{A5}$$

$$\tilde{x}(t) = x(t) + \mathcal{N}(0,\, \sigma^2_{\text{noise}}) \tag{A6}$$

where $\epsilon = 10^{-12}$ prevents division by zero. For DH signals, noise is added after min-max normalization (main text Eq. 3). Each SNR level uses an independent random number generator seeded as $\text{seed}_l = \text{seed}_{\text{base}} + l \times 1000$ (where l is the SNR level index), ensuring independent noise realizations across levels while maintaining within-level reproducibility. The same parameter configurations are used across all SNR levels and the clean condition, so that performance differences are attributable solely to noise. Each noise condition contains 70 training, 10 validation, and 20 test instances, mirroring the clean splits with identical parameter configurations.

Table A3 SNR levels for noise robustness evaluation. SNR 0 is the clean baseline with no noise added. SNR 1 through SNR 6 represent progressively increasing corruption.

SNR level	SNR (dB)	Description	Clinical analogue
0	∞ (clean)	No noise added	Ideal / ground truth
1	40	Near-clean	Controlled laboratory recording
2	30	Very low noise	Stationary bedside monitoring
3	20	Low noise	Ambulatory recording, minimal movement
4	10	Moderate noise	Ambulatory recording, routine activity
5	5	High noise	Wearable during exercise
6	1	Severe noise	Electrode displacement, heavy artifact

A2.2 Frequency distribution shift

To quantify out-of-distribution generalization, the carrier frequency range used during training is treated as shift-0. Additional test sets are generated from frequency bands systematically displaced from the training range. Given a training frequency interval $(f_{\text{low}}, f_{\text{high}})$ with width $w = f_{\text{high}} - f_{\text{low}}$:

- **Below training range**: the interval $[0, f_{\text{low}})$ is divided into $n_{\text{below}} = 2$ equally spaced sub-intervals, producing two buckets progressively further below the training distribution.
- **Above training range**: $n_{\text{above}} = 2$ additional intervals of width w are placed by stepping upward from f_{high}, i.e. bucket k spans $[f_{\text{low}} + kw,\ f_{\text{high}} + kw]$ for $k = 1, 2$.

This yields five frequency buckets in total. The frequency ranges for all three signal families are reported in Table A4. Within each out-of-distribution bucket, 20 test

signals are generated with the carrier frequency sampled uniformly from the bucket range and all non-frequency parameters (A, β, f_{mod}, c) sampled from the original training bounds. This design isolates the effect of frequency shift from other parameter variations.

Table A4 Frequency distribution shift setup for the three signal families. Shift -2 and -1 denote lower-frequency ranges, Shift 0 is the training distribution, and Shift $+1$ and $+2$ denote higher-frequency ranges. All values in Hz.

**Family**	**Shift -2**	**Shift -1**	**Shift 0 (Train)**	**Shift $+1$**	**Shift $+2$**
Drift-Harmonic	[0.35, 0.60]	[0.60, 0.85]	[0.85, 1.10]	[1.10, 1.35]	[1.35, 1.60]
SPM-Harmonic	[0.00, 0.34]	[0.34, 0.68]	[0.68, 1.41]	[1.41, 2.14]	[2.14, 2.88]
DPM-Harmonic	[0.00, 0.34]	[0.34, 0.68]	[0.68, 1.41]	[1.41, 2.14]	[2.14, 2.88]

A2.3 Single state transition

A deterministic frequency change-point is placed at a variable position t^* within the signal, testing how quickly models adapt to abrupt state changes once they become observable. The signal alternates between two frequency states whose ranges are deliberately separated to ensure identifiability:

$$f_0 \sim \mathcal{U}\big(f_{\mathrm{low}} + 0.05\,\Delta f,\;\; f_{\mathrm{low}} + 0.25\,\Delta f\big), \quad f_1 \sim \mathcal{U}\big(f_{\mathrm{low}} + 0.55\,\Delta f,\;\; f_{\mathrm{low}} + 0.75\,\Delta f\big) \tag{A7}$$

where $\Delta f = f_{\mathrm{high}} - f_{\mathrm{low}}$ is the span of the training frequency range. For SPM with $(f_{\mathrm{low}}, f_{\mathrm{high}}) = (0.6782, 1.4112)$, this yields $f_0 \in [0.71, 0.94]$ Hz and $f_1 \in [1.07, 1.21]$ Hz. The same separation logic is applied to modulation frequencies:

$$f_{\mathrm{mod},0} \sim \mathcal{U}\big(f_{\mathrm{mod}}^{\mathrm{low}}{+}0.05\,\Delta f_{\mathrm{mod}},\;\; f_{\mathrm{mod}}^{\mathrm{low}}{+}0.30\,\Delta f_{\mathrm{mod}}\big),\; f_{\mathrm{mod},1} \sim \mathcal{U}\big(f_{\mathrm{mod}}^{\mathrm{low}}{+}0.55\,\Delta f_{\mathrm{mod}},\;\; f_{\mathrm{mod}}^{\mathrm{low}}{+}0.90\,\Delta f_{\mathrm{mod}}\big) \tag{A8}$$

Small per-realization perturbations are added to amplitude and modulation depth to further differentiate states: $\Delta A \sim \mathcal{U}(0.01, 0.03)$ and $\Delta\beta \sim \mathcal{U}(0.02, 0.04)$.

Phase continuity.

To prevent artificial discontinuities at the change-point, which would introduce a non-physiological transient that models could exploit rather than genuinely adapting to the new frequency regime, the instantaneous phase is computed recursively:

$$\theta(k) = \theta(k-1) + 2\pi\, f_{S(k-1)}\,\Delta t, \quad k = 1, \ldots, T-1 \tag{A9}$$

where $S(k) \in \{0, 1\}$ is the state at sample k, $f_{S(k)}$ is the corresponding carrier frequency, and $\Delta t = 1/f_s$. This ensures that the phase accumulates smoothly across state boundaries, mimicking the continuous phase evolution of real physiological oscillators during state transitions.

Change-point placement.

The change-point t^* is sampled uniformly over $[0.25\,T,\ 0.75\,T]$, where T is the total signal length. Given a history window of $H = 50$ samples and a prediction window of $P = 100$ samples, this placement allows the transition to fall either within the observed history (in-context: positions H-2 through H-40 samples before the forecast boundary) or within the unobserved future (no-context: positions F-2 through F-40 samples after the forecast boundary). Each split contains 600 training, 100 validation, and 200 test instances.

A2.4 Markov switching

Signals alternate stochastically between two frequency states governed by a symmetric two-state Markov chain, simulating the probabilistic state dynamics that characterize cardiac rhythm variability, sleep-stage cycling, and autonomic fluctuations. At each time step, the state transitions with probability p or persists with probability $1 - p$:

$$S(k) = \begin{cases} 1 - S(k-1) & \text{with probability } p \\ S(k-1) & \text{with probability } 1 - p \end{cases} \tag{A10}$$

with $S(0) = 0$. This is evaluated at five transition probabilities: $p \in \{0.10, 0.30, 0.50, 0.70, 0.90\}$, spanning infrequent switching ($p = 0.10$) to near-continuous alternation ($p = 0.90$). Per-state frequency and modulation parameter ranges follow the well-separated protocol (Eq. A7). Modulation depth is additionally perturbed between states: $\beta_1 = \beta_0 + \Delta\beta$ with $\Delta\beta \sim \mathcal{U}(0.02, 0.04)$. Phase continuity is maintained via recursive accumulation (Eq. A9). For each value of p, the dataset contains 70 training, 10 validation, and 20 test instances (100 per p; 500 total across all five conditions). Signals are sampled at $f_s = 10$ Hz for 300 s ($T = 3{,}000$ samples).

A3 Forecasting Model Architectures and Hyperparameters

The main text benchmarks 11 forecasting models across four architectural families. This section provides architectural descriptions and full hyperparameter specifications for each family. All models share a unified training protocol (AdamW optimizer, OneCycleLR scheduling, MSE loss, batch size 128, early stopping on validation loss) unless noted otherwise in the per-family tables. Hyperparameters across different signal families were largely consistent, with only minor adjustments to learning rate and weight decay. Each model was trained independently on each signal type and evaluation paradigm.

A3.1 Linear models

Three linear models serve as computational efficiency baselines. **Linear** applies a single linear layer mapping from the input sequence to the prediction horizon.**DLinear** [8] decomposes the input into trend and seasonal components using a moving average

kernel before applying separate linear transformations to each component. **FITS** [26] operates in the complex frequency domain, interpolating low-frequency components to generate forecasts. All three models use Reversible Instance Normalization (RevIN) to handle distribution shift.

Table A5 Training hyperparameters for linear-family models.

**Hyperparameter**	**Linear**	**DLinear**	**FITS**
Training epochs	300	300	300
Learning rate	0.0001	0.0001	0.0001
Weight decay	0.001	0.001	0.001
Batch size	128	128	128
Patience	70	70	70
LR schedule	OneCycleLR	OneCycleLR	OneCycleLR
RevIN	Yes	Yes	Yes
Decomposition kernel	–	25	–
Cutoff frequency	–	–	15 Hz

A3.2 MLP-based models

Three MLP-based models introduce nonlinear transformations. **MLinear** is a two-layer multilayer perceptron with hidden dimensions [256, 512] and dropout regularization, serving as a nonlinear baseline. **N-BEATS** [27] (Neural Basis Expansion Analysis for Time Series) introduces a deep architecture with backward and forward residual links organized into stacks of fully connected blocks. Each block produces both a backcast (reconstruction of the input) and a forecast, enabling interpretable decomposition. N-BEATS is the only model in our benchmark that produces an explicit backcast output. **FreMLP** (FreTS) [28] is a frequency-domain MLP that operates in two stages: domain conversion, which maps time-domain signals into complex-valued frequency components via FFT, and frequency learning, where redesigned MLPs jointly learn the real and imaginary parts of these components.

A3.3 CNN-based models

Two CNN-based architectures process temporal structure through convolutional kernels with localized temporal processing windows. **ModernTCN** [20] is a temporal convolutional architecture featuring depthwise separable convolutions, residual connections, and structural reparameterization that fuses large and small kernels during inference to capture both short- and long-range temporal dependencies. **MICN** [19] (Multi-scale Isometric Convolution Network) employs a multi-branch structure: local features are extracted through downsampling convolutions, while global dependencies are modeled using isometric convolutions with linear complexity in sequence length. We evaluate both **MICN_Mean** and **MICN_Regre**, which implement different strategies for handling trend-cyclical components: MICN_Mean uses the mean

Table A6 Architectural and training hyperparameters for MLP-based models.

Hyperparameter	MLinear	N-BEATS	FreMLP
Number of layers	2	5 per block	2
Number of blocks	–	6	–
Hidden dimensions	256, 512	256, 512	256
Embed size	–	–	128
Activation	GELU	ReLU	ReLU
Block type	–	Generic	–
Backcast	No	Yes	No
MLP dropout	0.3	0.3	0.3
Weight decay	0.0001	0.0001	0.0001
Learning rate	0.0001	0.0001	0.0001
Training epochs	300	300	300
Patience	30	30	30
Batch size	128	128	128
LR schedule	OneCycleLR	OneCycleLR	OneCycleLR

of the decomposed trend for prediction, while MICN_Regre applies a regression-based approach.

A3.4 Transformer-based models

Three transformer variants represent the range from global to local attention. A standard **Transformer** adapted for time series forecasting with an encoder-decoder architecture serves as the baseline. **Autoformer** [24] replaces the self-attention mechanism with an auto-correlation module to capture long-range periodic dependencies and incorporates series decomposition within the architecture. **PatchTST** [18] divides the input time series into patches and applies transformer encoders over these patch-level representations, enabling localized attention patterns that preserve within-patch periodicity. For Transformer and Autoformer, half of the history window (25 time steps) was used as the label length to warm up the decoder. PatchTST uses 3 encoder layers (compared to 2 for the other transformer variants) and does not require a decoder.

A4 Fidelity Metric Computation

The main text defines three fidelity metrics (amplitude error, frequency error, and phase error) and their clinical rationale. This section provides full algorithmic details, including preprocessing steps, edge case handling, reliability filtering, and masking procedures needed for reproduction. All metrics are computed per-sequence over the H-step forecast horizon (excluding the history window), and per-sequence values are aggregated via the median when comparing models.

Table A7 Architectural and training hyperparameters for CNN-based models.

Hyperparameter	ModernTCN	MICN
Number of blocks	[2, 2, 2, 2]	–
Large kernel sizes	[21, 19, 17, 13]	–
Small kernel sizes	[3, 3, 3, 3]	–
Embedding dims	[64, 128, 256, 512]	–
FFN ratio	4	–
Patch size / stride	20 / 10	–
Conv kernels	–	[7, 17]
Decomposition kernels	–	[25, 49]
Isometric kernels	–	[17, 49]
Hidden dimensions	–	256, 512
Label length	–	50
Trend prediction mode	–	Regre / Mean
Dropout	0.2	–
Head dropout	0.1	–
MLP dropout	–	0.3
Learning rate	0.001	0.0001
Weight decay	0.001	0.0001
Training epochs	300	300
Patience	30	30
Batch size	128	128
LR schedule	OneCycleLR	OneCycleLR

A4.1 Amplitude error (MAE)

For each forecast sequence, amplitude error is computed as the mean absolute error between the predicted and true values over the prediction horizon:

$$\mathrm{MAE}_i = \frac{1}{H}\sum_{t=1}^{H}\left|\,\hat{y}_i(t) - y_i(t)\,\right| \tag{A11}$$

where i indexes the sequence and $H = 100$ is the prediction length. No normalization or scaling is applied; all signals share the same amplitude range by construction.

A4.2 Frequency error

Frequency error quantifies the mismatch in dominant oscillation rate between the predicted and true signals. The estimation proceeds in four steps.

Step 1: DC removal.

The signal mean is subtracted to eliminate the zero-frequency component: $x(t) \leftarrow x(t) - \bar{x}$.

Table A8 Architectural and training hyperparameters for transformer-based models.

Hyperparameter	PatchTST	Autoformer	Transformer
Encoder layers	3	2	2
Attention heads	8	8	8
Embed dimension	256	256	256
Feed-forward dim	256	256	256
Dropout	0.2	0.2	0.2
FC dropout	0.2	0.2	0.2
Head dropout	0.2	0.2	0.2
Patch length	15	–	–
Stride	10	–	–
Label length	–	25	25
Factor	–	–	3
RevIN	Yes	Yes	Yes
Decomposition	No	No	No
Training epochs	300	300	300
Patience	30	30	30
Learning rate	0.0001	0.0001	0.0001
Weight decay	0.0001	0.0001	0.0001
Batch size	128	128	128
LR schedule	OneCycleLR	OneCycleLR	OneCycleLR

Step 2: Power spectrum.

The one-sided power spectrum is computed via the real-valued FFT:

$$X(k) = \mathrm{FFT}_{\mathrm{real}}(x), \qquad P(k) = |X(k)|^2, \qquad k = 0, 1, \ldots, \lfloor N/2 \rfloor \tag{A12}$$

where N is the FFT length (equal to the signal length; no zero-padding is applied for frequency estimation).

Step 3: Peak detection with parabolic refinement.

The bin k^* with maximum power (excluding the DC bin $k = 0$) is identified. For non-edge bins ($1 < k^* < \lfloor N/2 \rfloor$), the frequency estimate is refined using three-point parabolic interpolation:

$$\delta = \frac{P(k^*-1) - P(k^*+1)}{2\big(P(k^*-1) - 2\,P(k^*) + P(k^*+1)\big)}, \qquad \hat{f} = (k^* + \delta)\,\frac{f_s}{N} \tag{A13}$$

where f_s is the sampling rate. This provides sub-bin accuracy without increasing the FFT length. For edge bins ($k^* = 1$ or $k^* = \lfloor N/2 \rfloor$), the unrefined bin-center estimate $\hat{f} = k^* \cdot f_s/N$ is used.

Step 4: Reliability filtering.

An estimate is marked as unreliable (NaN) and excluded from downstream analysis if either of the following conditions holds:

- **Low total power:** $\sum_k P(k) < 10^{-8}$ (effectively a flat or constant signal).
- **Diffuse spectrum:** $P(k^*) < 0.10 \cdot \sum_k P(k)$ (no single frequency dominates; the signal lacks a clear periodicity).

Both thresholds are applied identically to the true and predicted signals. If both estimates are reliable, the per-sequence frequency error is $\Delta f_i = |\hat{f}_{\text{pred},i} - \hat{f}_{\text{true},i}|$. If either estimate is unreliable, Δf_i is set to NaN and excluded. For model-level comparisons, only sequences where all models under comparison have finite frequency error are retained (intersection-valid masking), ensuring that pairwise tests are conducted on identical sample sets.

A4.3 Phase error

Phase error quantifies temporal misalignment between the predicted and true signals. The computation involves constructing the analytic signal, extracting instantaneous phase, and averaging the phase difference over reliable regions.

Step 1: Preprocessing.

The signal mean is subtracted: $x(t) \leftarrow x(t) - \bar{x}$.

Step 2: Analytic signal via frequency-domain Hilbert transform.

The analytic signal $z(t)$ is constructed by:

1. Zero-pad the signal to length $N_{\text{fft}} = 2N$ (pad factor = 2) to reduce circular convolution edge effects.
2. Compute the FFT: $X(k) = \text{FFT}(x_{\text{padded}})$.
3. Construct the one-sided spectral mask:

$$H(k) = \begin{cases} 1 & k = 0 \\ 2 & 1 \leq k < N_{\text{fft}}/2 \\ 1 & k = N_{\text{fft}}/2 \\ 0 & k > N_{\text{fft}}/2 \end{cases} \tag{A14}$$

4. Inverse transform and crop to the original length: $z(t) = \text{IFFT}(X \cdot H)\big|_{t=0}^{N-1}$.

The real part of $z(t)$ approximates the original signal, and the imaginary part is its Hilbert transform. This implementation is equivalent to `scipy.signal.hilbert` but provides explicit control over the padding factor. The pad factor of 2 was chosen to minimize edge artifacts; increasing it further did not meaningfully change the phase estimates on our signal families.

Step 3: Instantaneous phase extraction.

The instantaneous phase is extracted and unwrapped for temporal continuity:

$$\varphi(t) = \text{unwrap}\big(\arg(z(t))\big) \tag{A15}$$

Unwrapping removes 2π discontinuities, producing a monotonically evolving phase suitable for computing differences.

Step 4: Amplitude-based masking.

Phase estimates are unreliable where the signal amplitude is low (e.g., near zero crossings of a modulated signal). A binary mask selects only time points where the true signal has sufficient amplitude:

$$\mathcal{M} = \left\{\, t : |z_{\text{true}}(t)| > \alpha \cdot \text{median}\big(|z_{\text{true}}|\big) \,\right\}, \qquad \alpha = 0.2 \tag{A16}$$

The threshold $\alpha = 0.2$ (20% of the median instantaneous amplitude) was chosen to exclude low-amplitude regions while retaining the majority of the signal. If the median amplitude is zero or non-finite, or if no time points pass the mask, the sequence is marked NaN and excluded.

Step 5: Phase difference and wrapping.

The phase difference is computed at each masked time point and wrapped to $(-\pi, \pi]$:

$$\Delta\varphi(t) = \text{wrap}_\pi\big(\varphi_{\text{pred}}(t) - \varphi_{\text{true}}(t)\big), \qquad \text{wrap}_\pi(\theta) = \big((\theta + \pi) \bmod 2\pi\big) - \pi \tag{A17}$$

Step 6: Per-sequence phase error.

The per-sequence metric is the mean absolute wrapped phase difference over the mask, converted to degrees:

$$\Delta\varphi_i = \frac{180}{\pi} \cdot \frac{1}{|\mathcal{M}|} \sum_{t \in \mathcal{M}} |\Delta\varphi(t)| \tag{A18}$$

As with frequency error, intersection-valid masking is applied for model-level comparisons.

A4.4 Residual variance analysis at comparable MAE

To quantify the extent to which MAE fails to resolve fidelity differences across architectures, we defined a *comparable-MAE window* for each signal family as the central 60% of median-MAE values across the 11 models (20th–80th percentile of MAE). Within this window, models are, by construction, effectively indistinguishable by conventional pointwise accuracy. For each architecture family (CNN, MLP, Linear-family, Transformer) we then computed the mean phase error and the mean frequency error over the family's models whose MAE fell inside the window. The dissociation Δ reported in Table A9 and marked by the red arrow in main-text Fig. 3 is the gap between the highest and lowest family mean in a given panel, quantifying the fidelity spread that MAE alone cannot detect.

Table A9 Family-level fidelity at comparable MAE. The comparable-MAE window is the central 60% (20th–80th percentile) of MAE values across the 11 models. Entries are mean fidelity error per architecture family over models inside the window; per-family model counts in parentheses. Δ is the gap between the highest and lowest family mean in each row and corresponds to the red arrow in main-text Fig. 3. DH = Drift Harmonic; SPM = Single-Phase Modulation; DPM = Dual-Phase Modulation. Linear-family models account for the upper extreme of both metrics in all three signal families.

Metric	Family	MAE Window	CNN	MLP	Linear	Transf.	Δ
Phase error (°)	DH	[0.003, 0.059]	1.62 (3)	–	29.48 (2)	2.14 (2)	27.87
	SPM	[0.011, 0.064]	7.20 (2)	12.83 (1)	53.30 (2)	33.42 (2)	46.10
	DPM	[0.020, 0.081]	9.68 (1)	58.87 (2)	62.28 (3)	9.80 (1)	52.59
Frequency error (Hz)	DH	[0.003, 0.059]	0.0002 (3)	–	0.0080 (2)	0.0002 (2)	0.0078
	SPM	[0.011, 0.064]	0.0004 (2)	0.0008 (1)	0.0192 (2)	0.0158 (2)	0.0189
	DPM	[0.020, 0.081]	0.0020 (1)	0.0411 (2)	0.0475 (3)	0.0015 (1)	0.0460

A5 Statistical Analysis

A5.1 Paired testing

For each fidelity metric, we computed paired differences between each model and the Linear baseline on a per-sequence basis. For clean, noise, and frequency shift paradigms, significance was assessed using the paired t-test:

$$t = \frac{\bar{d}}{s_d/\sqrt{n}}, \qquad p = 2\big(1 - \Phi(|t|)\big) \tag{A19}$$

where $\bar{d}$ is the mean paired difference, s_d the standard deviation, n the number of paired sequences, and Φ the standard normal CDF. 95% confidence intervals were computed as $\bar{d} \pm 1.96 \cdot s_d/\sqrt{n}$.

For state-transition analyses, where error distributions are non-Gaussian due to the mixture of in-context and no-context conditions, we used the Wilcoxon signed-rank test with tie correction. Zero differences are removed, the absolute values of remaining differences are ranked (with average ranks assigned to ties), and the sum of ranks for positive differences W^+ is computed. The z-statistic with tie-corrected variance is:

$$z = \frac{W^+ - \mu}{\sqrt{\sigma^2}}, \qquad \mu = \frac{n(n+1)}{4}, \qquad \sigma^2 = \frac{n(n+1)(2n+1)}{24} - \sum_g \frac{t_g^3 - t_g}{48} \tag{A20}$$

where t_g is the number of ties in group g. Two-sided p-value: $p = 2(1 - \Phi(|z|))$.

A5.2 Multiple comparison correction

All p-values within each metric and paradigm were adjusted using the Holm step-down procedure:

1. Sort the m raw p-values in ascending order: $p_{(1)} \leq p_{(2)} \leq \cdots \leq p_{(m)}$.

2. Multiply each by its rank-dependent factor: $\tilde{p}_{(k)} = (m - k + 1) \cdot p_{(k)}$.
3. Enforce monotonicity: $p_{(k)}^{\text{Holm}} = \max\big(\tilde{p}_{(k)}, p_{(k-1)}^{\text{Holm}}\big)$, capped at 1.0.

The Holm procedure controls the family-wise error rate at $\alpha = 0.05$ while providing uniformly greater power than the classical Bonferroni correction.

A5.3 Intersection-valid masking

For frequency and phase error, spectral reliability filtering (§A4.2, §A4.3) can produce NaN values for individual sequences. To ensure that all models are compared on exactly the same set of sequences, we apply intersection-valid masking: a sequence is included in the comparison only if all models under evaluation have a finite (non-NaN) value for that metric on that sequence. This prevents differences in sample composition from confounding pairwise comparisons.

A5.4 State-transition adaptation analysis

For the state-transition paradigm, sequences are grouped by distance tags indicating how far the transition lies from the forecast boundary. Tags take the form H-XX (transition is XX timesteps before the boundary, within the observable history) and F-XX (transition is XX timesteps after the boundary, in the unobserved future). The distance bins are $XX \in \{2, 4, 6, 10, 12, 15, 20, 30, 40\}$. Two additional tags, A and B, denote sequences with no transition in the evaluation window, serving as steady-state baselines for each frequency state.

Within each tag, a Wilcoxon signed-rank test is performed comparing each model to the Linear baseline, with Holm correction applied separately per tag. Adaptation speed is characterized by the first history tag at which a model's median phase error drops below a clinically meaningful threshold of 20°. For a digital twin receiving data at 10 Hz, this provides a direct translation from timesteps to seconds of delayed state detection. For example, a model reaching 20° at tag $H - 6$ requires approximately 0.6 seconds of post-transition context, while one reaching 20° at tag $H - 40$ requires 4.0 seconds.

A5.5 Markov fidelity assessment (HMM proxy)

The HMM-based evaluation assesses whether forecasting models preserve the temporal structure of stochastic state switching. The procedure is as follows:

1. **Feature extraction.** Extract the dominant frequency from each window using a Welch periodogram (window = 16 samples, hop = 8 samples, $f_s = 10$ Hz).
2. **Normalization.** Z-score normalize features across all sequences.
3. **HMM fitting.** Fit a two-state Gaussian HMM on the true-history features, selecting the best model across eight random seeds (0, 1, 2, 3, 4, 5, 10, 20) by log-likelihood.
4. **State canonicalization.** Relabel states so that state 0 always has the lower emission mean.
5. **Decoding.** Decode state sequences for both true-future and predicted-future using the fitted HMM.

6. **Switching probability.** Compute the windowed switching probability (flip rate) for each decoded sequence.
7. **Distributional fit.** Fit Gaussian distributions to the switching-probability distributions of true-history.
8. **Comparison.** Compare distributions via symmetric KL divergence:

$$\mathrm{KL}_{\mathrm{sym}} = \mathrm{KL}(P\|Q) + \mathrm{KL}(Q\|P) \tag{A21}$$

where, for univariate Gaussians:

$$\mathrm{KL}(P\|Q) = \ln \frac{\sigma_Q}{\sigma_P} + \frac{\sigma_P^2 + (\mu_P - \mu_Q)^2}{2\,\sigma_Q^2} - \frac{1}{2} \tag{A22}$$

A model is classified as capturing the switching dynamics at a given transition probability if $\mathrm{KL}_{\mathrm{sym}} < 0.05$. Sensitivity to this threshold is analyzed in §A6.5.

A5.6 Pareto frontier construction

Models were scored across five evaluation paradigms: clean accuracy, noise robustness, shift robustness, state-transition adaptation, and Markov fidelity. Within each paradigm, scores were computed as the aggregate improvement over the Linear baseline (averaged across signal families and fidelity metrics), then min-max normalized to $[0, 1]$ across models so that a score of 1.0 corresponds to the best-performing model on that paradigm and 0.0 to the worst.

A model A is said to dominate model B if A scores greater than or equal to B on all five paradigms and strictly greater on at least one. The Pareto frontier consists of all non-dominated models. Models not on the frontier are classified as dominated. The full normalized scores and Pareto classifications are reported in Table A11.

A6 Extended Results

This section reports extended results across signal families and evaluation paradigms. The structure mirrors the order in which the analyses are referenced from the main text: clean-condition fidelity profiles (§A6.1), noise robustness across signal families (§A6.2), frequency-shift robustness across signal families (§A6.3), state-transition adaptation across fidelity dimensions (§A6.4), Markov switching with threshold sensitivity (§A6.5), and multi-paradigm performance profiles (§A6.6).

A6.1 Three-dimensional fidelity profiles under clean conditions

The main text reports phase fidelity as the primary diagnostic for evaluating architectural suitability, as phase preservation showed the largest dissociation from conventional MAE and the clearest architectural separation. Here we present the full three-dimensional fidelity profiles, covering phase, frequency, and amplitude improvement over the linear baseline, for all 11 architectures across all three signal families (Figs. A1–A3). These results demonstrate that the architectural hierarchy observed

for phase fidelity extends consistently to frequency and amplitude dimensions, with informative differences in magnitude and ranking that validate the need for separate diagnostics.

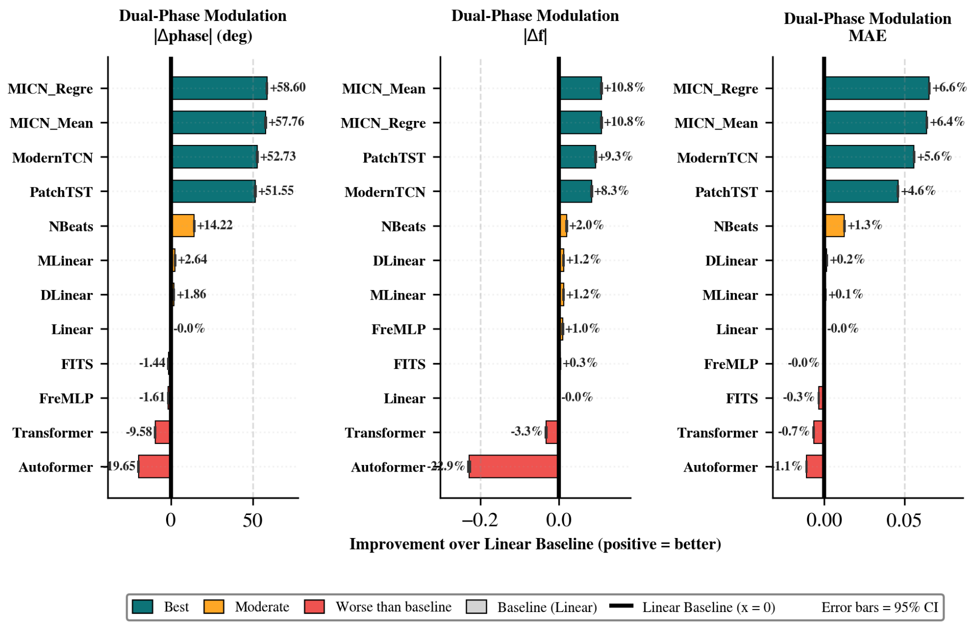

Fig. A1 Full fidelity profile for dual-phase modulation signals under clean conditions. Phase improvement (|Δphase|, degrees; left), frequency improvement ($|\Delta f|$; center), and amplitude improvement (MAE; right) over the linear baseline for all 11 architectures. MICN variants lead all three dimensions: MICN_Mean and MICN_Regre achieve +10.8% frequency improvement and +6.4% to +6.6% amplitude improvement alongside $> 57°$ phase gains. PatchTST and ModernTCN follow closely in frequency (+9.3% and +8.3%) and amplitude (+4.6% and +5.6%). Transformer and Autoformer degrade across all three dimensions (Transformer: $-9.58°$ phase, -3.3% frequency, -0.7% amplitude; Autoformer: $-19.65°$ phase, -22.9% frequency, -1.1% amplitude), confirming that their phase failure reflects a broader inability to preserve dynamical structure rather than an isolated timing deficit. Error bars represent 95% confidence intervals. Color denotes performance tier: best (teal), moderate (orange), baseline (gray), worse than baseline (red).

On dual-phase modulation signals (Fig. A1), the most spectrally complex family, the top-performing architectures maintained their advantage across all three fidelity dimensions. MICN variants achieved the highest frequency improvement (+10.8% for both MICN_Mean and MICN_Regre) and amplitude improvement (+6.4% and +6.6%), consistent with their leading phase performance (+57.76° and +58.60°). PatchTST and ModernTCN followed in all three dimensions. Critically, Transformer and Autoformer degraded not only in phase but also in frequency (-3.3% and -22.9%) and amplitude (-0.7% and -1.1%), confirming that their failure is not limited to timing but extends to the full dynamical profile. Had evaluation relied on MAE alone, Autoformer's -22.9% frequency degradation would have been invisible.

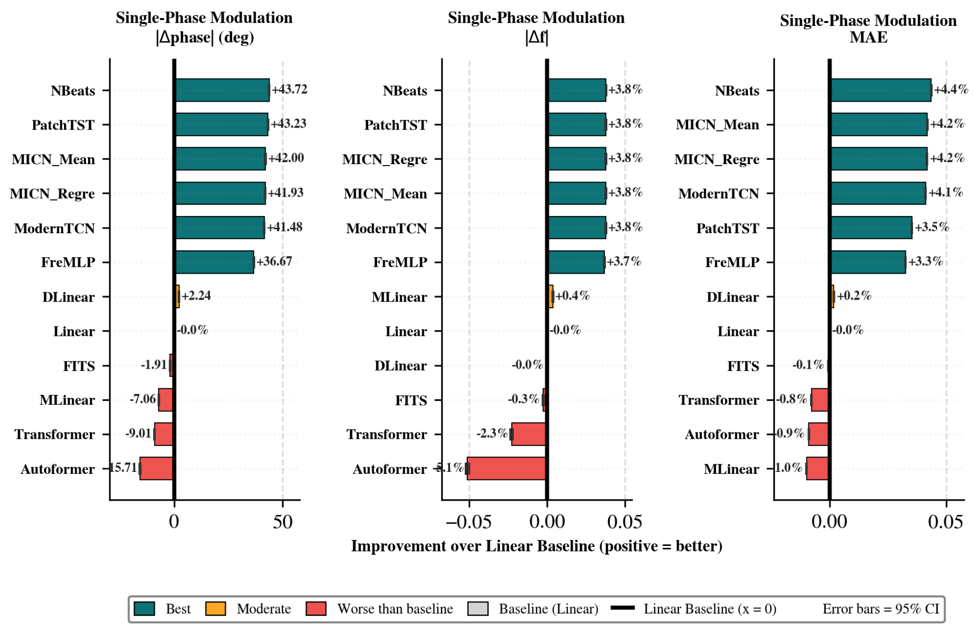

Fig. A2 Full fidelity profile for single-phase modulation signals under clean conditions. Phase improvement (left), frequency improvement (center), and amplitude improvement (right) over the linear baseline. The top tier compresses relative to dual-phase signals: NBeats (+43.72°), PatchTST (+43.23°), MICN_Mean (+42.00°), MICN_Regre (+41.93°), ModernTCN (+41.48°), and FreMLP (+36.67°) all achieve substantial phase improvement, with frequency improvements tightly clustered between +3.7% and +3.8% and amplitude improvements between +3.3% and +4.4%. MLinear shows a dimension-specific dissociation: moderate frequency improvement (+0.4%) alongside phase degradation (−7.06°), illustrating a failure mode that only separate fidelity diagnostics can detect. Transformer (−9.01° phase, −2.3% frequency, −0.8% amplitude) and Autoformer (−15.71° phase, −9.1% frequency, −0.9% amplitude) again degrade across all dimensions. Error bars represent 95% confidence intervals.

On single-phase modulation signals (Fig. A2), the architectural spread narrowed as the reduced spectral complexity placed lower demands on temporal processing window structure. Six architectures achieved phase improvements between +36° and +44°, with frequency improvements tightly clustered near +3.8% and amplitude improvements between +3.3% and +4.4%. The narrower separation is consistent with the signal-complexity interaction reported in the main text. A notable finding was the dimension-specific dissociation exhibited by MLinear: it achieved +0.4% frequency improvement and +1.2% phase improvement on this signal family, but degraded to −7.06° in phase on the same signals, demonstrating that a model can preserve one fidelity dimension while failing on another. This is precisely the failure mode that separate diagnostics are designed to detect and that conventional MAE evaluation would miss entirely.

On drift-harmonic signals (Fig. A3), the simplest family, nearly all architectures improved over baseline across all three dimensions. NBeats led in phase (+53.50°),

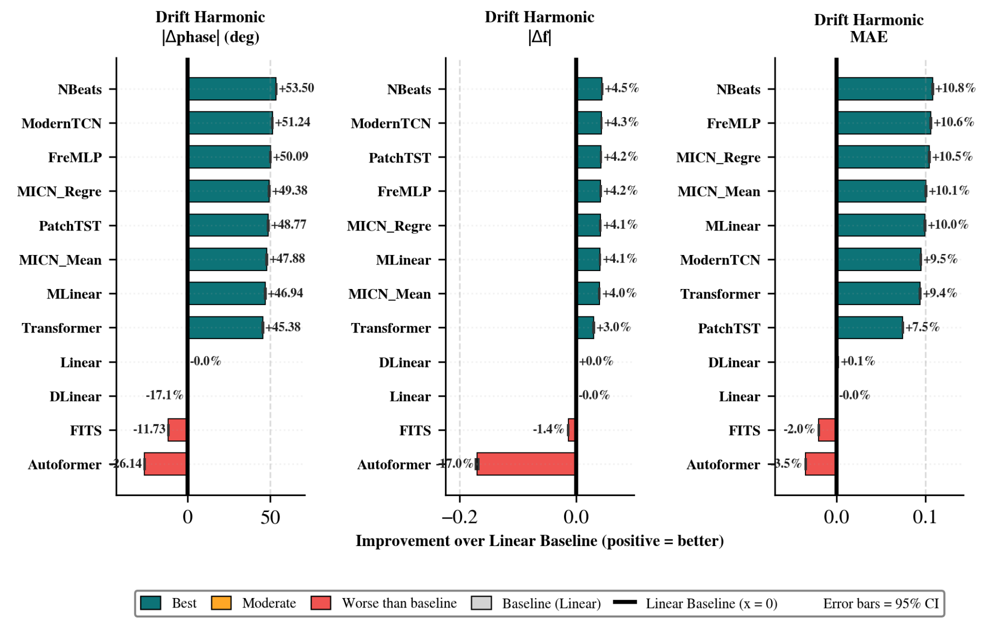

Fig. A3 Full fidelity profile for drift-harmonic signals under clean conditions. Phase improvement (left), frequency improvement (center), and amplitude improvement (right) over the linear baseline. Nearly all architectures improve over baseline across all three dimensions, with NBeats leading in phase (+53.50°), frequency (+4.5%), and amplitude (+10.8%). Transformer achieves strong performance across all dimensions (+45.38° phase, +3.0% frequency, +9.4% amplitude), confirming that global attention preserves dynamical structure when spectral complexity is low. Even on these simplest signals, Autoformer remains the weakest architecture (−26.14° phase, −17.0% frequency, −3.5% amplitude) and FITS degrades across all dimensions (−11.73° phase, −1.4% frequency, −2.0% amplitude), indicating fundamental mismatches to oscillatory signal preservation regardless of complexity. Error bars represent 95% confidence intervals.

frequency (+4.5%), and amplitude (+10.8%). The most informative finding was Transformer's strong three-dimensional performance (+45.38° phase, +3.0% frequency, +9.4% amplitude), which stands in sharp contrast to its degradation on dual-phase signals. This confirms that the Transformer's failure on complex signals is not a general architectural deficiency but a specific inability to handle multi-frequency modulation, a distinction that would be obscured by reporting performance on any single signal family alone.

A6.2 Noise robustness across signal families

The main text reports noise robustness on dual-phase modulation signals, the most spectrally complex family. Here we present the full noise robustness profiles for single-phase modulation (Fig. A4) and drift-harmonic (Fig. A5) signals, confirming that PatchTST and MICN variants consistently occupy the top tier across signal families despite shifts in their relative ordering.

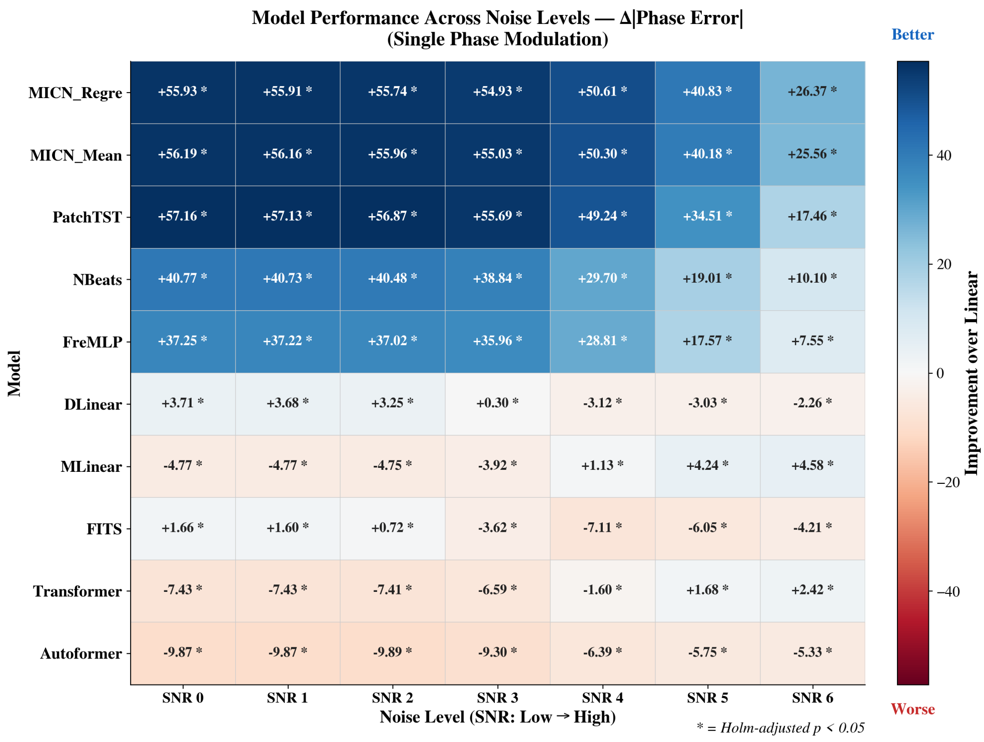

Fig. A4 Noise robustness for single-phase modulation signals. Phase improvement (Δ|phase|, degrees) over the linear baseline across seven noise levels (SNR 0 to SNR 6, where higher SNR number corresponds to more severe corruption). MICN_Regre and MICN_Mean lead across all noise levels, retaining +26.37° and +25.56° at SNR 6. PatchTST retains +17.46° at SNR 6, remaining in the top tier despite a steeper decline from +57.16° at SNR 0. Their relative ordering reverses compared with dual-phase signals (main-text Fig. 5), with MICN variants outperforming PatchTST under severe corruption, but both remain substantially ahead of all other architectures. MLinear exhibits a crossover from negative improvement at low noise (−4.77° at SNR 0) to positive at high noise (+4.58° at SNR 6), and Transformer shows a similar reversal (−7.43° to +2.42°), indicating that global mappings gain a relative advantage once noise obscures localized temporal structure, though their absolute improvement remains far below PatchTST and MICN. Autoformer remains the worst performer across all noise levels (−9.87° to −5.33°). All differences marked with ∗ are Holm-corrected $p < 0.05$.

On single-phase modulation signals (Fig. A4), MICN_Regre and MICN_Mean retained the strongest phase improvement across all noise levels, with MICN_Regre holding +55.93° at SNR 0 and +26.37° at SNR 6. PatchTST followed closely (+57.16° at SNR 0, +17.46° at SNR 6), with both architectures substantially ahead of NBeats (+40.77° to +10.10°) and FreMLP (+37.25° to +7.55°). The relative ordering between PatchTST and MICN reversed compared with dual-phase signals: MICN_Regre outperformed PatchTST at SNR 6 by +8.91° despite comparable clean-condition baselines, indicating that MICN's multi-scale convolution provides more stable retention under severe corruption on single-frequency signals. Two architectures exhibited informative crossovers: MLinear shifted from −4.77° at SNR 0 to +4.58°

at SNR 6, and Transformer shifted from $-7.43°$ to $+2.42°$, suggesting that global mappings gain a relative advantage once noise obscures the localized temporal structure that patch-based and convolutional models exploit. However, even at their best noise level these architectures remained far below the PatchTST and MICN tier. Autoformer remained the worst performer ($-9.87°$ to $-5.33°$), and FITS degraded from marginally positive ($+1.66°$) to consistently negative ($-4.21°$).

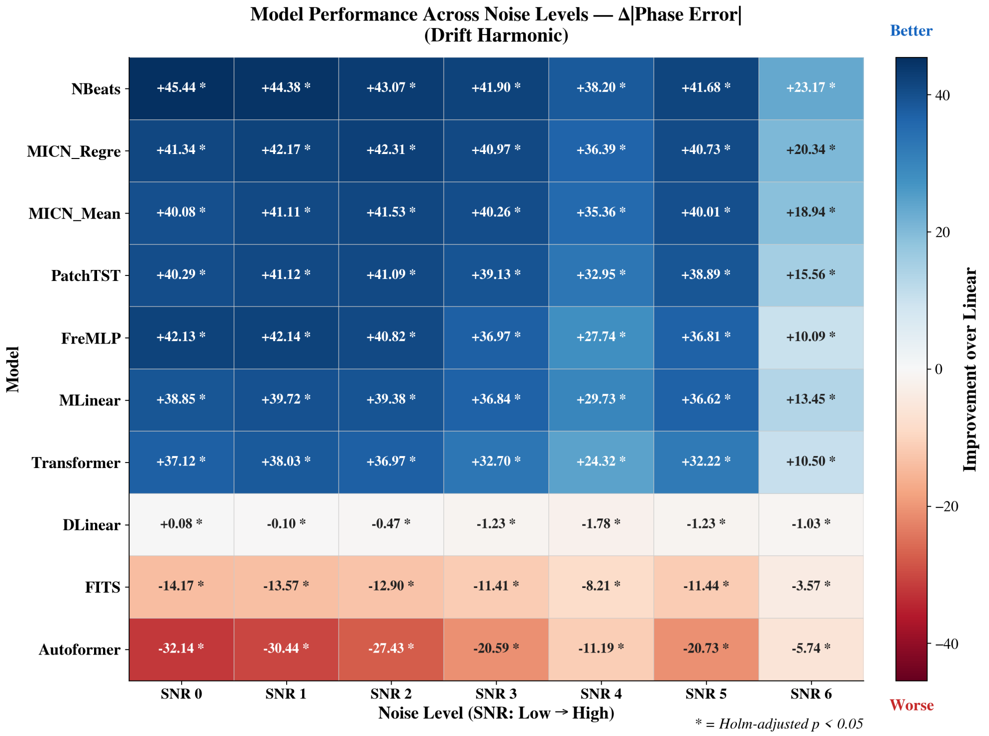

Fig. A5 Noise robustness for drift-harmonic signals. Phase improvement over the linear baseline across seven noise levels. NBeats leads at SNR 6 ($+23.17°$), with MICN_Regre ($+20.34°$), MICN_Mean ($+18.94°$), and PatchTST ($+15.56°$) close behind. The architectural spread compresses relative to more complex signal families, consistent with reduced demands on temporal processing window structure when only a single slowly varying frequency is present. Nearly all nonlinear architectures retain positive improvement across the full noise range, including Transformer ($+37.12°$ to $+10.50°$) and MLinear ($+38.85°$ to $+13.45°$). DLinear hovers near zero throughout. FITS ($-14.17°$ to $-3.57°$) and Autoformer ($-32.14°$ to $-5.74°$) remain the only architectures with consistently negative improvement. All differences marked with $*$ are Holm-corrected $p < 0.05$.

On drift-harmonic signals (Fig. A5), the gap between architectures compressed substantially as the simpler spectral structure reduced demands on temporal processing windows. NBeats showed the best retention at SNR 6 ($+23.17°$), followed by MICN_Regre ($+20.34°$), MICN_Mean ($+18.94°$), and PatchTST ($+15.56°$). Notably, nearly all nonlinear architectures retained positive improvement across the full noise

range, including Transformer(+37.12° to +10.50°) and MLinear (+38.85° to +13.45°), both of which had degraded on more complex signal families. FreMLP exhibited the steepest decline among top models(+42.13° to +10.09°), consistent with noise amplifying spectral leakage in frequency-domain representations. Even on these simplest signals, Autoformer remained the worst performer (−32.14° to −5.74°) and FITS showed consistent degradation (−14.17° to −3.57°).

Taken together, the cross-signal noise results reinforce two findings from the main text. First, PatchTST and MICN variants consistently occupied the top tier across all three signal families, with their relative ordering shifting (PatchTST leading on dual-phase, MICN leading on single-phase, NBeats leading on drift-harmonic at the highest noise) but both remaining substantially ahead of linear-family and global attention architectures. Second, the noise robustness hierarchy interacted with signal complexity: on spectrally complex signals, only PatchTST and MICN maintained strong improvement under noise, whereas on simpler signals a broader range of architectures retained fidelity.

A6.3 Frequency shift robustness across signal families

The main text reports frequency shift robustness on single-phase modulation signals. Here we present the full shift robustness profiles for dual-phase modulation (Fig. A6) and drift-harmonic (Fig. A7) signals, revealing that the severity of frequency shift degradation scales with signal complexity and that a directional asymmetry emerges on simpler signals.

On dual-phase modulation signals (Fig. A6), the collapse under frequency shift was substantially more severe than on single-phase signals. At Shift 0, the architectural hierarchy matched the clean-condition results: MICN_Regre (+58.60°), MICN_Mean (+57.76°), and PatchTST (+51.55°) led, with NBeats at +14.22°. Under any degree of shift, however, these advantages effectively disappeared. MICN_Regre fell to −4.57° at Shift −2 and −1.15° at Shift +2, PatchTST fell to −1.74° and −1.15°, and NBeats fell to −1.90° and −1.56°. The collapse was roughly symmetric across positive and negative shifts, and no architecture maintained more than +2° improvement at anyshifted condition. This contrasts with single-phase signals where PatchTST retained +8.85° at Shift −2, indicating that multi-frequency modulation amplifies sensitivity to distributional mismatch. DLinear showed modest but stable improvement across all shifts (+0.81° to +1.86°), consistent with its shift-invariant linear mapping. Transformer (−9.58° at Shift 0, −5.81° at Shift −2) and Autoformer (−19.65° at Shift 0) degraded even without shift, confirming their unsuitability for nonstationary monitoring regardless of shift severity.

On drift-harmonic signals (Fig. A7), a directional asymmetry emerged that was not apparent on the other signal families. At Shift 0, nearly all architectures achieved strong improvement, with NBeats (+53.50°), FreMLP (+50.09°), MICN_Regre (+49.38°), PatchTST (+48.77°), and MICN_Mean (+47.88°) all above +47°. Under negative shifts (lower frequencies at test time), degradation was substantially greater than under positive shifts. MICN_Mean fell to −10.32° at Shift −2 but only −1.94° at Shift +2. MICN_Regre showed a comparable pattern (−9.47° vs. +0.22°), and PatchTST followed the same trend (−8.33° vs. +1.60°). This directional

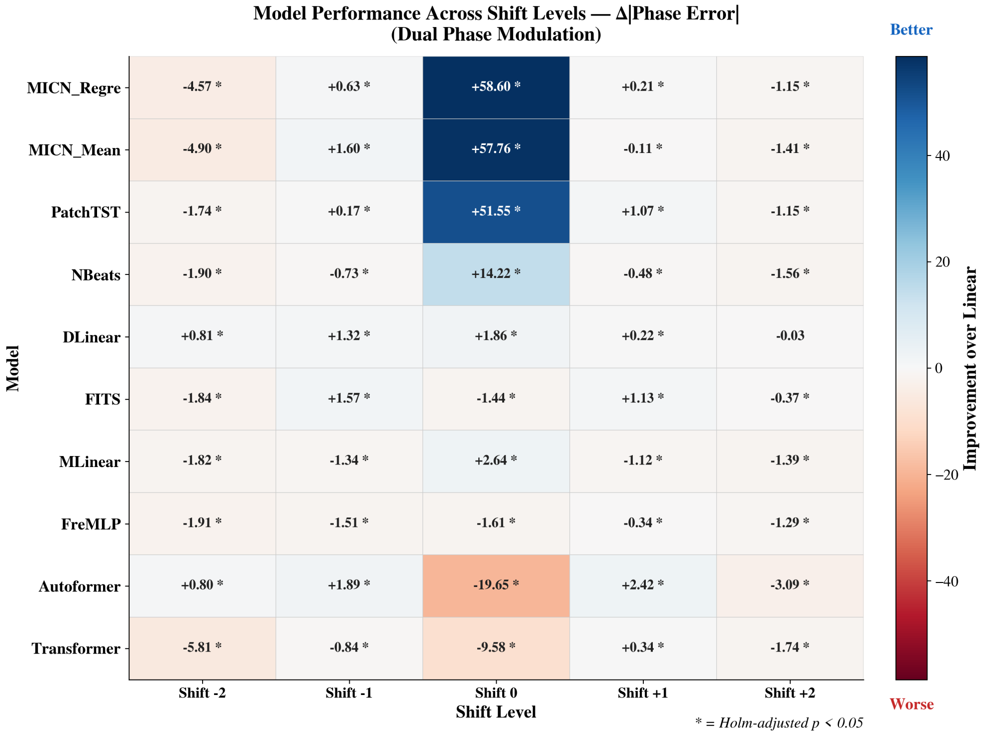

Fig. A6 Frequency shift robustness for dual-phase modulation signals. Phase improvement (Δ|phase|, degrees) over the linear baseline across five shift levels (Shift -2 to Shift $+2$). At Shift 0, MICN_Regre ($+58.60°$), MICN_Mean ($+57.76°$), and PatchTST ($+51.55°$) achieve strong improvement, consistent with clean-condition results. Under any frequency shift, these advantages collapse: MICN_Regre falls to $-4.57°$ at Shift -2 and $-1.15°$ at Shift $+2$, and PatchTST falls to $-1.74°$ and $-1.15°$. The collapse is roughly symmetric and more severe than on single-phase signals (main-text Fig. 6b), indicating that multi-frequency modulation amplifies sensitivity to distributional mismatch. NBeats ($+14.22°$ at Shift 0) also collapses under shift ($-1.90°$ at Shift -2, $-1.56°$ at Shift $+2$). DLinear shows modest but stable improvement across shifts ($+0.81°$ to $+1.86°$). Transformer ($-9.58°$) and Autoformer ($-19.65°$) degrade even at Shift 0. All differences marked with $*$ are Holm-corrected $p < 0.05$.

asymmetry suggests that downward frequency shifts, such as transitions from active to resting physiological states, pose a greater challenge than upward shifts for models trained on higher-frequency bands. Notably, Transformer ($+45.38°$ at Shift 0) also showed this asymmetry ($-5.45°$ at Shift -2 vs. $+1.19°$ at Shift $+2$), indicating that the effect is not architecture-specific but reflects a general property of how models extrapolate beyond their training frequency range. Autoformer remained the worst performer at Shift 0 ($-26.14°$) but paradoxically showed less degradation under shift ($-0.40°$ at Shift -2, $+4.53°$ at Shift $+2$), likely because its already-poor baseline left less room to degrade.

Taken together, the cross-signal shift results reinforce two findings from the main text. First, PatchTST and MICN variants maintained the strongest improvement at

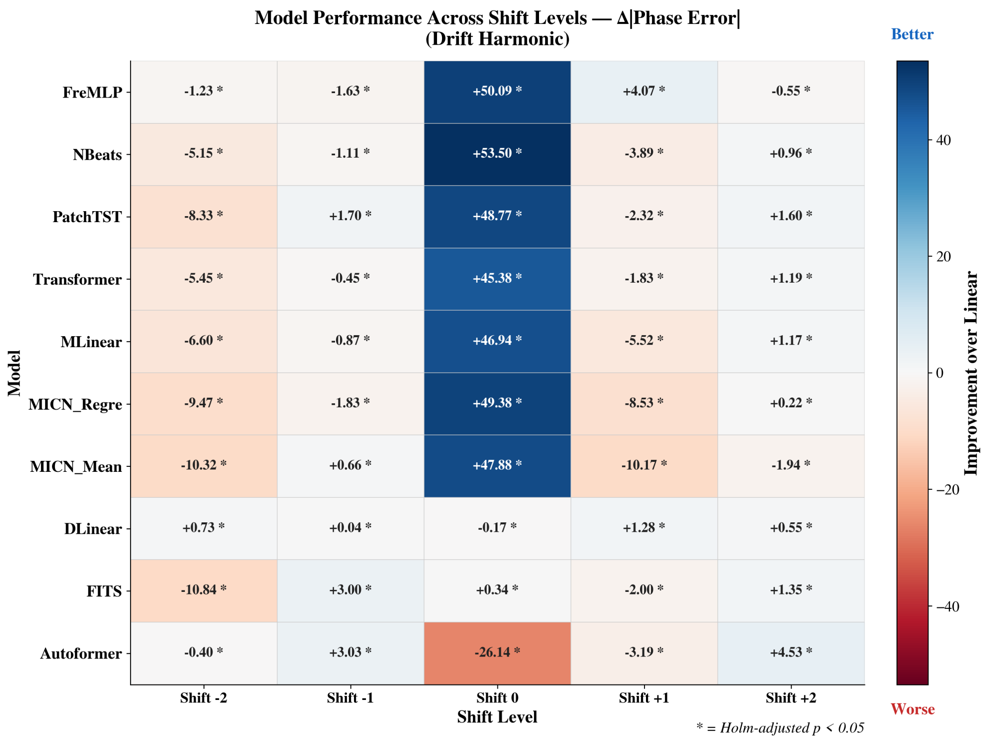

Fig. A7 Frequency shift robustness for drift-harmonic signals. Phase improvement over the linear baseline across five shift levels. At Shift 0, NBeats (+53.50°), FreMLP(+50.09°), MICN_Regre (+49.38°), PatchTST(+48.77°), and MICN_Mean (+47.88°) all achieve strong improvement. A directional asymmetry emerges under shift: negative shifts produce larger degradation than positive shifts across most architectures. MICN_Mean falls to $-10.32°$ at Shift -2 but only $-1.94°$ at Shift $+2$. MICN_Regre falls to $-9.47°$ at Shift -2 but retains $+0.22°$ at Shift $+2$. PatchTST falls to $-8.33°$ at Shift -2 but retains $+1.60°$ at Shift $+2$. This asymmetry suggests that downward frequency shifts, such as transitions from active to resting states, pose a greater challenge than upward shifts for models trained on higher-frequency bands. Autoformer ($-26.14°$ atShift 0) remains the worst performer. All differences marked with $*$ are Holm-corrected $p < 0.05$.

Shift 0 across all signal families, confirming their suitability as the default architectural choice for digital twins operating within their training distribution. Second, no architecture maintained substantial improvement beyond moderate shifts (± 1) on any signal family, and the severity of collapse increased with signal complexity. The directional asymmetry on drift-harmonic signals adds a new consideration: digital twins monitoring signals where downward frequency shifts are clinically relevant, such as transitions from active wakefulness to drowsiness in EEG, may require more frequent model updating than those tracking upward shifts.

A6.4 State transition adaptation across fidelity dimensions

The main text reports state transition adaptation speed using phase fidelity as the primary diagnostic, as phase preservation showed the clearest differentiation between architectural adaptation rates. Here we present the full tag-wise profiles for amplitude fidelity (MAE; Fig. A8) and frequency fidelity (Fig. A9) across all signal tags, confirming that the adaptation speed hierarchy identified through phase error extends consistently to the other two fidelity dimensions, with additional dimension-specific patterns.

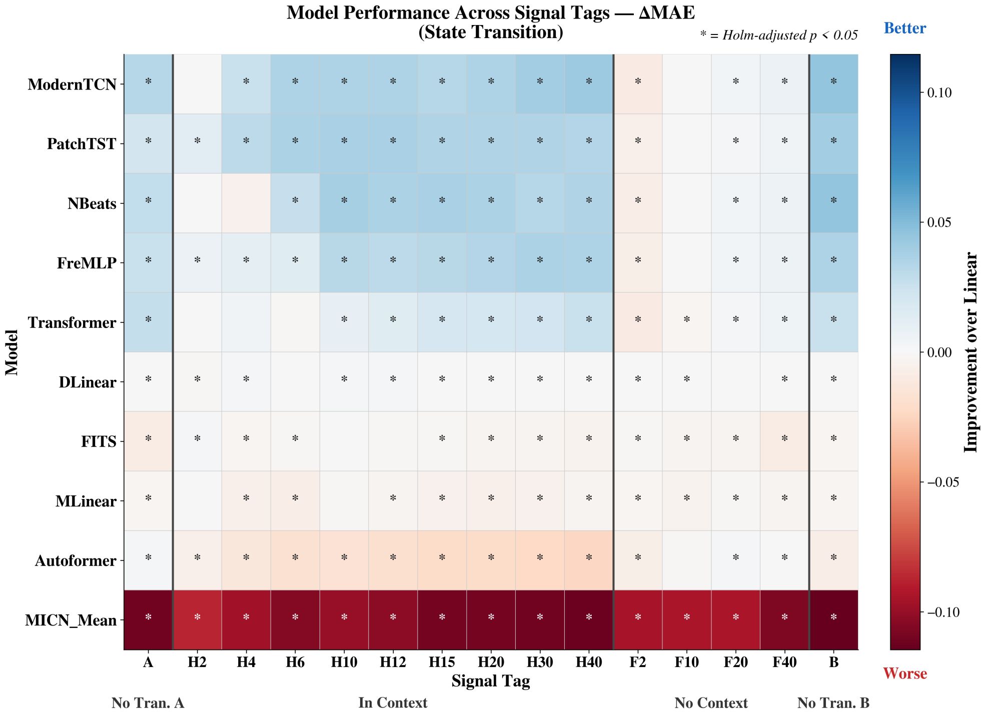

Fig. A8 Amplitude fidelity (MAE) across state transition tags. Improvement in MAE over the linear baseline for all 11 architectures across signal tags: no-transition baselines (A, B), in-context transitions (H2 to H40), and no-context transitions (F2 to F40). ModernTCN and PatchTST show the strongest and most consistent amplitude improvement across in-context tags, with improvement intensifying as more post-transition context becomes available. FreMLP shows consistent moderate improvement across all in-context tags. NBeats shows weaker amplitude adaptation than its phase adaptation, with modest improvement emerging only after H6. MICN_Mean shows consistent amplitude degradation across all tags (dark red), contrasting with its strong clean-condition amplitude performance and indicating that state transitions specifically disrupt its amplitude preservation. Autoformer shows degradation concentrated at in-context tags with moderate context (H6 to H40). Linear-family models (DLinear, MLinear, FITS) show mixed patterns with generally weak improvement. All differences marked with $*$ are Holm-corrected $p < 0.05$.

For amplitude fidelity (Fig. A8), the adaptation speed hierarchy broadly matched the phase results but with notable differences. ModernTCN and PatchTST showed the strongest and most consistent amplitude improvement across in-context tags, with improvement intensifying as more post-transition context became available, consistent with their rapid phase adaptation reported in the main text. FreMLP maintained consistent moderate improvement across all in-context tags. NBeats, which showed strong phase adaptation, exhibited weaker amplitude adaptation, with modest improvement emerging only after H6, suggesting that its basis expansion architecture recovers oscillatory timing before it recovers oscillation magnitude. The most striking finding was MICN_Mean's consistent amplitude degradation across all tags including no-transition baselines, shown as uniformly dark red in the heatmap. This contrasts with MICN_Mean's strong clean-condition amplitude performance (Fig. A1, +6.4%), indicating that the introduction of state transitions specifically disrupts MICN's amplitude preservation even when the transition itself has not yet occurred, possibly through sensitivity of its multi-scale decomposition to nonstationarity in the training distribution. When transitions occurred in the unobserved future (F2 to F40), amplitude improvement generally weakened across all architectures, paralleling the phase results.

For frequency fidelity (Fig. A9), a different pattern emerged that reveals dimension-specific adaptation dynamics. MICN_Mean achieved the strongest frequency improvement across in-context tags (H10 to H40), contrasting sharply with its amplitude degradation on the same tags. This dimension-specific dissociation, strong frequency preservation alongside amplitude degradation, is precisely the kind of failure mode that separate fidelity diagnostics are designed to detect: had evaluation relied on either metric alone, MICN_Mean would have appeared either excellent (frequency) or poor (amplitude) for state transition adaptation, when in reality it exhibits a complex, dimension-dependent profile. ModernTCN and PatchTST showed strong frequency improvement at early tags (H2), consistent with their rapid phase adaptation and confirming that localized temporal processing windows enable fast recovery across multiple fidelity dimensions simultaneously. NBeats showed an unexpected non-monotonic pattern: strong frequency improvement at H2 that diminished at intermediate tags before recovering at later tags, suggesting that its basis expansion architecture undergoes a transient frequency adjustment period during adaptation. Autoformer showed the largest frequency degradation across in-context tags, with dark red cells from H4 to H40, confirming its consistent unsuitability across all fidelity dimensions. MLinear exhibited a sharp frequency degradation specifically at H4, indicating a narrow window of instability during early adaptation.

These cross-dimensional profiles reinforce the main text finding that localized temporal processing architectures (PatchTST, ModernTCN) adapt fastest, while adding the nuance that adaptation speed can vary across fidelity dimensions within the same architecture. MICN_Mean's contrasting performance across frequency (strong) and amplitude (weak) during state transitions illustrates that architecture selection for digital twins monitoring state-change-prone signals should consider which fidelity dimension is most clinically relevant: if frequency tracking is paramount (as in seizure detection where frequency shifts precede generalization), MICN may be

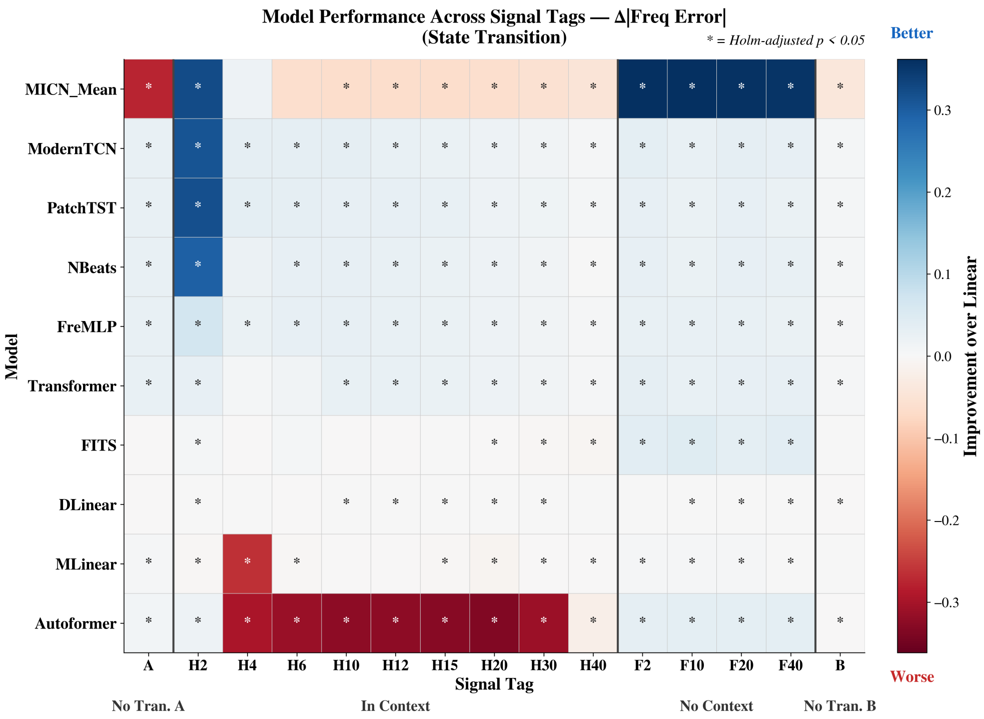

Fig. A9 Frequency fidelity across state transition tags. Improvement in frequency error ($\Delta|f|$) over the linear baseline across signal tags. MICN_Mean achieves the strongest frequency improvement across in-context tags (H10 to H40), with deep blue indicating substantial improvement over Linear. ModernTCN and PatchTST show strong frequency improvement at early tags (H2), consistent with their rapid phase adaptation. NBeats shows strong frequency improvement at H2 that diminishes at intermediate tags before recovering, suggesting non-monotonic frequency adaptation. Autoformer shows the largest frequency degradation across in-context tags (dark red, H4 to H40), and MLinear shows a sharp frequency degradation at H4. Transformer shows weak frequency adaptation throughout. In the no-context condition (F2 to F40), MICN_Mean retains substantial frequency improvement while most other architectures show moderate improvement, indicating that MICN's frequency preservation is more robust to unseen transitions than its amplitude preservation. All differences marked with $*$ are Holm-corrected $p < 0.05$.

suitable despite its amplitude vulnerability, whereas if amplitude preservation is critical (as in hemodynamic monitoring), PatchTST or ModernTCN offer more balanced adaptation.

A6.5 Stochastic switching: full results and threshold sensitivity

The main text reports that stochastic switching was the most challenging paradigm across all 11 architectures, with PatchTST succeeding at 2 of 5 transition probabilities and five architectures failing entirely. Here we present the complete KL divergence

table across all models and transition probabilities (Table A10), and a sensitivity analysis confirming that the architectural ranking is robust to threshold choice (Fig. A10).

Full KL divergence results.

Table A10 reports the symmetric KL divergence between true-history and predicted-future state-emission distributions for all 11 architectures across five transition probabilities. No model achieved KL < 0.05 at low transition probabilities ($p = 0.10$ or $p = 0.30$), where states persist for long durations and switching events are rare within any given window. All passes occurred at $p \geq 0.50$, where more frequent switching provides sufficient within-window evidence of alternation.

Table A10 Symmetric KL divergence across all models and transition probabilities. Lower values indicate closer distributional match. Bold values fall below the 0.05 threshold used for pass/fail classification in the main text. Models are ordered by pass count (descending), then by mean KL.

**Model**	$p = 0.10$	$p = 0.30$	$p = 0.50$	$p = 0.70$	$p = 0.90$
PatchTST	0.209	0.234	0.063	**0.008**	**0.046**
ModernTCN	0.573	0.584	0.187	**0.014**	0.062
MICN_Mean	0.742	0.186	**0.016**	0.108	0.114
MICN_Regre	0.743	0.175	**0.026**	0.096	0.114
FreMLP	0.399	1.154	**0.028**	0.061	0.073
DLinear	0.618	1.061	0.261	0.278	**0.022**
Linear	0.911	1.094	0.342	0.309	**0.034**
NBeats	0.324	0.061	0.121	0.085	0.066
Transformer	0.795	1.242	0.137	0.102	0.160
FITS	0.574	0.291	0.276	0.375	0.202
MLinear	0.319	1.362	1.180	1.347	0.094
Autoformer	1.007	0.521	1.371	1.460	2.020

Several patterns in the full table merit attention. PatchTST achieved the lowest KL values at $p = 0.70$ (0.008) and $p = 0.90$ (0.046), with both successes occurring at higher transition probabilities where the model receives more within-window evidence of alternation. At lower probabilities ($p = 0.10$ and $p = 0.30$), where states persist for longer durations and switches are rare, PatchTST failed (KL $= 0.209$ and 0.234), indicating that even the best-performing architecture requires sufficient switching events within its temporal processing window to encode the transition structure. ModernTCN passed only at $p = 0.70$ (KL $= 0.014$) but narrowly missed at $p = 0.90$ (KL $= 0.062$), suggesting that its convolutional temporal processing window is tuned to a narrow range of switching rates. MICN_Mean and MICN_Regre both passed at $p = 0.50$ (KL $= 0.016$ and 0.026) but failed elsewhere, and FreMLP passed at $p = 0.50$ (KL $= 0.028$) but showed highly inconsistent recovery across neighboring probabilities ($p = 0.30$: KL $= 1.154$; $p = 0.70$: KL $= 0.061$), revealing erratic rather than gradual degradation. DLinear and Linear passed only at $p = 0.90$ (KL $= 0.022$ and 0.034),

the highest switching rate, where the signal approaches rapid alternation that can be captured as an averaged pattern.

NBeats is a notable case: despite strong performance on the deterministic state-transition paradigm (recovering phase within 15 timesteps in the main text), it showed no capacity to recover stochastic switching (KL range: 0.061 to 0.324), with its closest approach at $p = 0.30$ (KL = 0.061) narrowly missing the threshold. This dissociation highlights that fast deterministic adaptation and probabilistic state recovery are distinct capabilities. Autoformer showed the worst overall performance, with KL exceeding 0.5 at every probability level (range: 0.521 to 2.020), indicating systematic divergence from the true switching distribution. MLinear failed broadly (KL range: 0.094 to 1.362), and Transformer showed moderate but consistently above-threshold divergence (KL range: 0.102 to 1.242).

Sensitivity to KL threshold.

The pass/fail classification in the main text uses a symmetric KL threshold of 0.05. To assess whether the architectural ranking depends on this specific choice, we evaluated all models at four thresholds: 0.05, 0.10, 0.15, and 0.20 (Fig. A10).

At the strictest threshold (KL < 0.05), PatchTST passed at 2 of 5 probability levels, six models at 1/5, and five models at 0/5. Relaxing to KL < 0.10 promoted FreMLP, NBeats, and PatchTST to 3/5, with NBeats' near-miss at $p = 0.30$ (KL $= 0.061$) now classified as a pass. Autoformer, FITS, and Transformer remained at 0/5. At KL < 0.15, PatchTST reached 3/5 and ModernTCN reached 2/5. At the most lenient threshold (KL < 0.20), PatchTST and NBeats reached 4/5, but Autoformer remained at 0/5 (KL range: 0.521 to 2.020). The rank ordering was preserved across all four thresholds: PatchTST consistently occupied the top position, the 1/5 models formed a stable middle tier, and Autoformer, FITS, and Transformer consistently occupied the bottom tier. This stability confirms that the main-text finding, that stochasticswitching dynamics remain largely unrecoverable by current forecasting architectures, is robust to the specific threshold used for classification.

A6.6 Multi-paradigm performance profiles

The main text summarizes multi-paradigm performance via the Pareto frontier (main-text Fig. 9). To provide a more detailed view of each architecture's strengths and weaknesses, Fig. A11 overlays all 11 models on a single radar chart, and Table A11 reports the full normalized scores and Pareto classifications.

Each axis represents one of the five evaluation paradigms (clean accuracy, noise robustness, shift robustness, state-transition adaptation, and Markov fidelity). Scores are min-max normalized across models (0 to 1 scale), where higher values indicate better performance relative to the other architectures.

The normalized scores reveal several patterns not immediately visible in the Pareto frontier visualization. NBeats, despite being classified as dominated, achieved the third-highest mean score (0.85) with no dimension below 0.78, making it a strong general-purpose alternative when balanced performance is prioritized over frontier optimality. The gap between NBeats and the frontier is narrow: it is dominated by PatchTST across all five dimensions but only marginally so on clean (0.82 vs 0.97) and

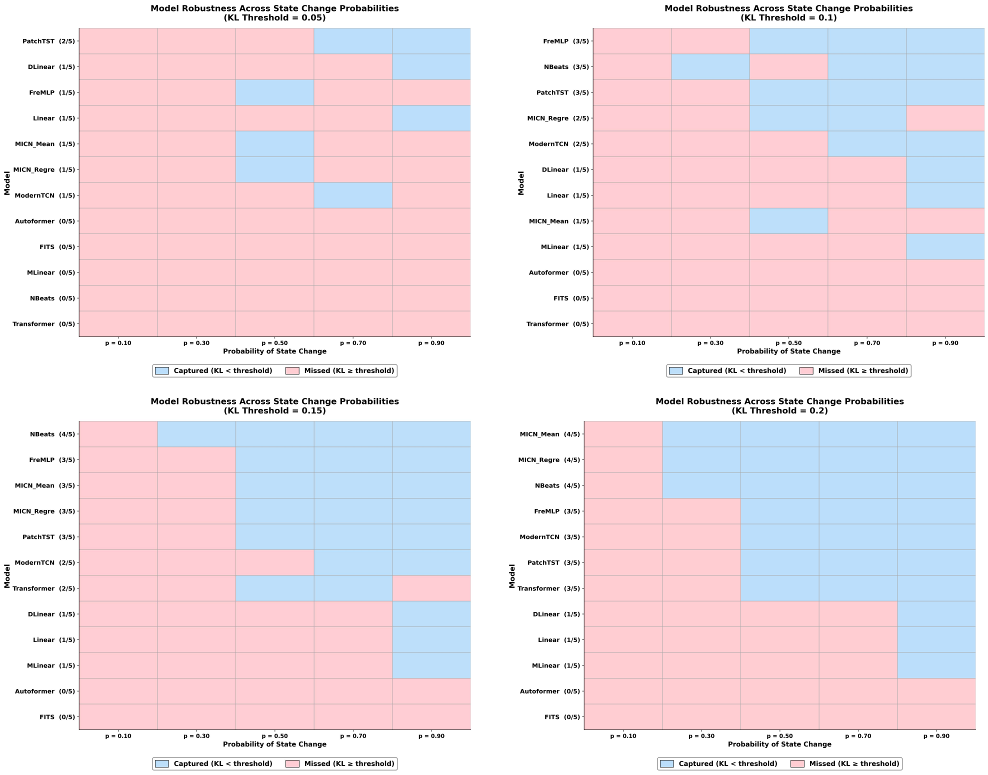

Fig. A10 Pass/fail classification is robust to KL threshold choice. Each panel shows the model × transition-probability pass/fail matrix at a different symmetric KL threshold. Blue: KL below threshold (captured); red: KL at or above threshold (missed). Models are sorted by pass rate (descending). At KL < 0.05 (main-text threshold), PatchTST leads with 2/5. At KL < 0.10, FreMLP, NBeats, and PatchTST each reach 3/5. At KL < 0.20, PatchTST and NBeats reach 4/5. Autoformer remains at 0/5 across all thresholds (KL range: 0.521 to 2.020). The rank ordering is stable across all fourthresholds, confirming that the main-text conclusions are not an artifact of a single threshold choice.

noise (0.84 vs 0.98). MICN_Mean's state-transition score of 0.00 reflects its consistent degradation below linear baseline on that paradigm, a vulnerability that would be masked by its excellent clean and noise scores (both 0.99) if only aggregate performance were reported. ModernTCN's frontier status depends entirely on its state-transition strength (0.97), as its noise (0.31) and shift (0.23) scores are among the lowest, illustrating how Pareto optimality can reflect narrow specialization rather than balanced capability. Autoformer scored 0.00 on clean accuracy, noise robustness, and Markov fidelity, confirming its unsuitability for any digital twin application evaluated in this study.

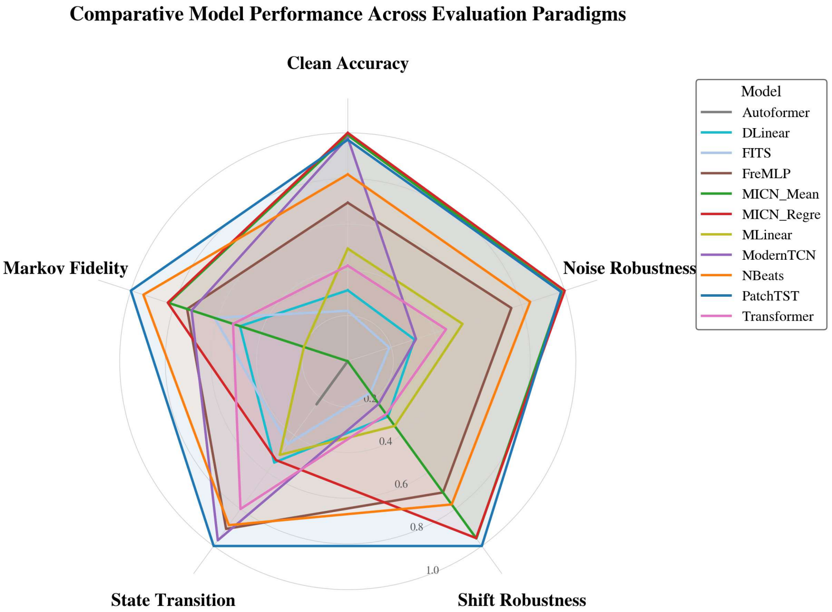

Fig. A11 Comparative model performance across all five evaluation paradigms. Radar chart overlaying all 11 models. PatchTST (blue) is the most balanced model and leads on three of five axes.NBeats (orange) shows consistently high performance but is narrowly dominated by PatchTST. MICN variants (green) achieve the highest clean accuracy and noise robustness but collapse on state-transition adaptation. Linear-family models (cyan/light blue) and Autoformer (gray) occupy the interior, indicating weak performance across most paradigms. Scores are min-max normalized across models per paradigm (0 to 1 scale).

Table A11 Normalized paradigm scores and Pareto classification. Scores are min-max normalized (0 to 1) across models per paradigm. Frontier models cannot be improved on any dimension without sacrificing another. PatchTST achieves the highest mean score (0.99) and is the only model above 0.95 on all five dimensions. MICN variants score highest on clean and noise but are weakened by state-transition performance. Autoformer scores 0.00 on three of five dimensions.

Model	Clean	Noise	Shift	State Tr.	Markov	Mean	Status
PatchTST	0.97	0.98	1.00	1.00	1.00	0.99	Frontier
MICN_Regre	1.00	1.00	0.96	0.54	0.83	0.86	Frontier
NBeats	0.82	0.84	0.78	0.89	0.94	0.85	Dominated
MICN_Mean	0.99	0.99	0.96	0.00	0.82	0.75	Frontier
FreMLP	0.69	0.75	0.71	0.91	0.74	0.76	Dominated
ModernTCN	0.98	0.31	0.23	0.97	0.72	0.64	Frontier
Transformer	0.42	0.45	0.29	0.80	0.53	0.50	Dominated
MLinear	0.49	0.53	0.35	0.51	0.20	0.42	Dominated
DLinear	0.31	0.31	0.30	0.55	0.50	0.39	Dominated
FITS	0.22	0.19	0.17	0.45	0.61	0.33	Dominated
Autoformer	0.00	0.00	0.00	0.23	0.00	0.05	Dominated
